# Hypoxia-Induced Degenerative Protein Modifications Associated with Aging and Age-Associated Disorders

**DOI:** 10.14336/AD.2019.0604

**Published:** 2020-03-09

**Authors:** Sunil S Adav, Siu Kwan Sze

**Affiliations:** ^1^School of Biological Sciences, Nanyang Technological University, Singapore; ^2^Singapore Phenome Centre, Lee Kong Chian School of Medicine, Nanyang Technological University, Singapore

**Keywords:** protein aggregation, hypoxia, aging, neurodegenerative disease, dementia, proteostasis, proteomics

## Abstract

Aging is an inevitable time-dependent decline of various physiological functions that finally leads to death. Progressive protein damage and aggregation have been proposed as the root cause of imbalance in regulatory processes and risk factors for aging and neurodegenerative diseases. Oxygen is a modulator of aging. The oxygen-deprived conditions (hypoxia) leads to oxidative stress, cellular damage and protein modifications. Despite unambiguous evidence of the critical role of spontaneous non-enzymatic Degenerative Protein Modifications (DPMs) such as oxidation, glycation, carbonylation, carbamylation, and deamidation, that impart deleterious structural and functional protein alterations during aging and age-associated disorders, the mechanism that mediates these modifications is poorly understood. This review summarizes up-to-date information and recent developments that correlate DPMs, aging, hypoxia, and age-associated neurodegenerative diseases. Despite numerous advances in the study of the molecular hallmark of aging, hypoxia, and degenerative protein modifications during aging and age-associated pathologies, a major challenge remains there to dissect the relative contribution of different DPMs in aging (either natural or hypoxia-induced) and age-associated neurodegeneration.

## 1. Introduction

Aging is nature’s most complex phenomena, where an inevitable time-dependent progressive functional decline of numerous physiological functions finally leads to death. These changes, either programmed or due to protein damage, lead to an imbalance in a regulatory system including hormones, repair, immune and neuroendocrine. Many theories of aging have been proposed [1]. The theory of longevity believes switching “on” and “off” of certain genes [2, 3]. The endocrine theory considers hormones control biological clocks [1]. While the immune theory recommends the programmed decline of immunity over time [4]. Of numerous damage theories. i.e. wear and tear, cross-linking and free radical theory; the majority of them are centered on protein damages through cross-linking, oxidative modifications by reactive oxygen species (ROS) or reactive nitrogen species (RNS) [1]. In short, aging is a consequence of two independent biological processes, one programmed loss of functionality and other damage related changes [1-3]. The damage-related changes include protein aggregation, structural modifications, loss of resistance to stress, and failure to repair the damages. According to an aging theory proposed by Harman in 1956 [5], the alterations in biological function with time is due to the accumulation of damages caused by free radicals.

**Figure 1.**
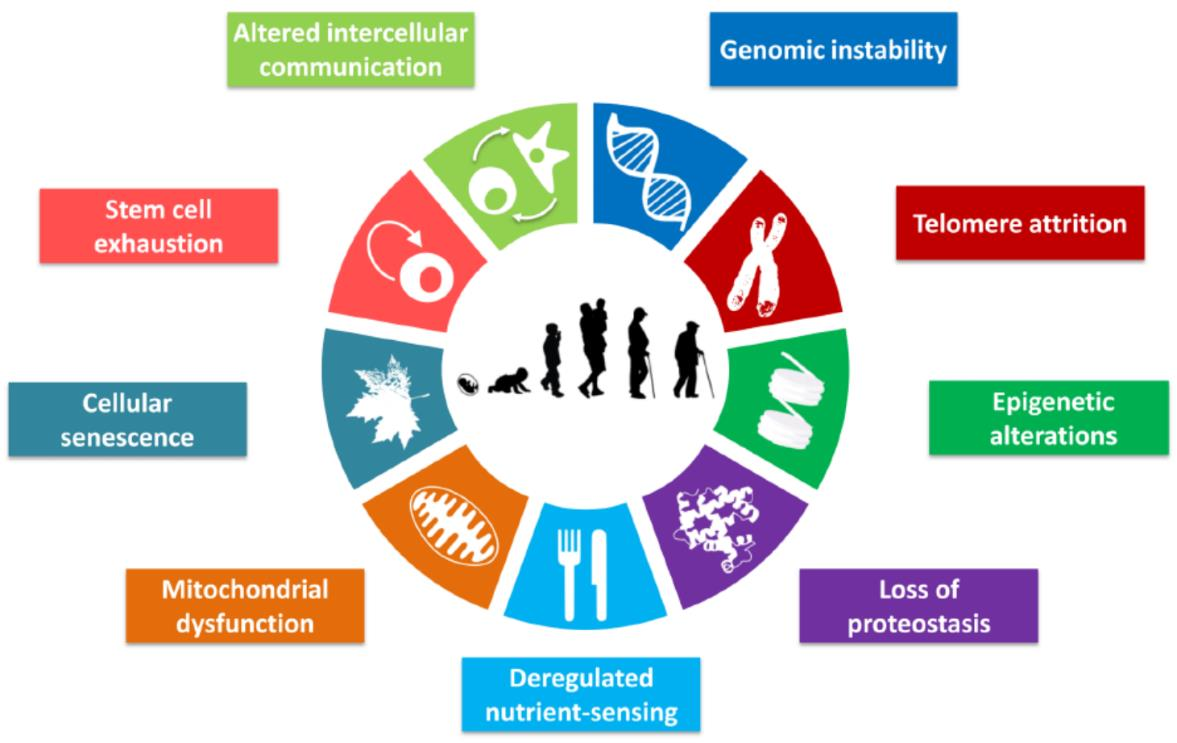
Schematics of nine hallmark of aging (adopted from López-Otín et al.[6]). The figure specifies the nine hallmarks of aging such as genomic instability, telomere attrition, epigenetic alterations, loss of proteostasis, deregulated nutrient-sensing, mitochondrial dysfunction, cellular senescence, stem cell exhaustion, and altered intercellular communication.

It is not clear how the loss of physiological integrity with cumulative dysfunction occurs in cells, and which factors are responsible for aging and unique aspects increase susceptibility to diseases, respectively. Basically, proteins, carbohydrates, lipids, and nucleic acids are four fundamental molecules without which cells and organisms cannot be living, hence they are named “molecules of life”. Aging of these molecules cannot be ignored since they are susceptible to free radical attack and actively involved in tissue aging. Recently, Lopez-Otin et al., [6] reviewed the recent literature and proposed nine tentative hallmarks of aging including genomic instability, epigenetic alterations, loss of proteostasis, telomere attrition, deregulated nutrient sensing, mitochondrial dysfunction, cellular senescence, stem cell exhaustion, and altered intercellular communication (Fig. 1). In all nine hallmarks of aging, proteins remains as a key component e.g. proteins stabilize genome through dynamic modification of chromatin architecture [7], epigenetic alterations are regulated by histones [8], proteostasis and mitochondrial functions are governed by proteins, and DNA-damages are repaired by proteins, and damaged DNA are finally translated into proteins. Thus, the protein damage could be crucial in aging and pathogenesis.

Proteins are the building blocks of life as they are not only the structural constituents of the living organisms but also a final functional molecule governing most of the biological functions. The proteins undergo alterations by spontaneous non-enzymatic Degenerative Protein Modifications (DPMs) including oxidation, deamidation, carbamylation, carbonylation, glycation etc. The DPMs change protein charge state, hydrophobicity and three-dimensional structure that influence functional activities and induce aggregation [9-15]. These protein modifications and accumulation of modified proteins are allied to aging and the development of age-associated pathologies like neurodegenerative diseases [10, 16]. DPMs like spontaneous protein deamidation characterized by the modification of glutaminyl and asparaginyl residues were hypothesized as a molecular timer of biological events including protein turn over, development and aging [17-20]. Protein deamidation progressively disrupts structural integrity of the protein and alter their biological activity [12, 13, 21]. Other DPMs including glycation, advanced glycation end products, oxidation, carbonylation, carbamylation, etc., impart deleterious structural and functional changes in proteins and impair their normal function [12, 13, 22, 23].

**Figure 2.**
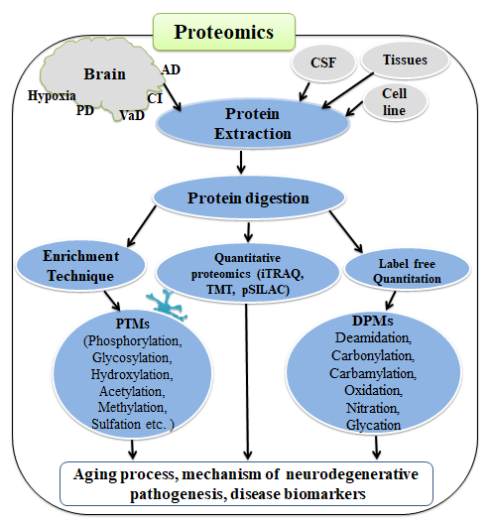
Advanced novel proteomic approaches to elucidate degenerative protein modifications (DPMs) and post-translational modifications (PTMs) of protein in aging, hypoxia and neurodegenerative diseases or other biological samples. (VaD: vascular dementia, CSF: cerebrospinal fluid, CI: cerebral ischemia, AD: Alzheimer disease, PD: Parkinson disease)

Hypoxia, a condition where oxygen supply to tissue is inadequate, induces free radical generation leading to oxidative protein modifications and tissue damage [24-27]. Oxygen supply also acts as a modulator of aging processes [28]. The cerebrovascular disorders and hypoxia-ischemia injuries in the brain are projected as a primary cause of protein pathologies that leads to cognitive impairment and dementia [29, 30]. In short, hypoxia-ischemia injury in the brain persuades DPMs that can lead to aging, age-associated diseases and neurodegeneration. The mechanisms that initiate and promote DPMs remain poorly understood, partly due to technical challenges. However, mass spectrometry not only has the potential to identify and quantify proteins but also identify DPMs quantitatively (Fig. 2). Therefore, the main purpose of this review was to present the recent advances in DPMs research in aging, hypoxia, and neurodegenerative diseases. Given the complexity of DPMs and their role in aging and age-related neurodegenerative diseases, we highlighted current discoveries of DPMs and their impacts on various cellular processes. We attempted to identify the linkage between hypoxia-induced protein modifications and dysfunctions, the aging process, and age-related neurodegenerative diseases.

## 2. Hypoxia

### 2.1. Correlation between hypoxia, aging and neurodegenerative diseases

The regular function of virtually all tissues and organs depends on adequate blood flow and oxygen supply. Any condition that results in insufficient blood flow leads to acute or chronic hypoxia and numerous complications including diminished metabolic processes, compromised neuronal signaling [31], cellular damages and functional impairments in the affected tissue[32]. Based on the oxygen level in tissue, hypoxia is classified as physoxia (5% oxygen), physiological hypoxia (2%), pathological hypoxia (1%), radiobiological hypoxia (0.4%) and anoxia (0%) [33]. The short-term exposure to hypoxia, ischemia, hyperoxia, hyperthermia, and hypothermia may enhance the adaptive mechanism by overcoming stress, and remains beneficial [34, 35]. In mild hypoxia, the oxygen sensing system stimulates gene expression including hypoxia-inducible transcription factor-1α (HIF-1α), which in turn controls the expression of survival genes to protect the tissue from damages[36]. It’s important to note that the arterial blood oxygen concentration remains at 9.5% (95000 ppm) and the cellular damage begins to occur when it drops below 5000 ppm oxygen [37].

Patients with obstructive sleep apnea, Cheyne-stokes respiration, and nocturnal hypoventilation experience chronic intermittent hypoxia (CIH) more often than continuous hypoxia [38]. CIH induces modification of the proteins involved in transcriptional activation, signaling pathways, transmitter synthesis and cardioprotection [39]. The decrease in air pressure and air density with increase in altitude (during aviation) result in hypobaric hypoxia, which causes neurodegeneration and memory impairment in animal models [40, 41]. Chronic cerebral ischemia induced by cerebral hypoperfusion in a bilateral carotid artery stenosis mouse model damaged brain tissue, intercellular communication and caused impairment of working memory and neurovascular unit dysfunction observed in early Alzheimer's disease [42, 43]. Biswal and colleagues [44] noted increased neurodegeneration with altered mitochondrial morphology and aggregation of lipofuscin granules in the hippocampus region. These authors also found an elevated level of pro-inflammatory S100A9 protein and reduced expression of synaptosome associated protein 25 (SNAP25), total *Tau*, superoxide dismutase 2 (SOD2), and apolipoprotein E (ApoE) in young rats when exposed to chronic global hypoxia. These findings correlate hypoxia and neurodegeneration with aging in the hippocampus. Further, neurodegeneration and dendritic atrophy in the hippocampus acts as a contributing factor for spatial memory impairment upon chronic exposure to hypobaric hypoxia [41]. The magnitude of deleterious effects increases with a decrease in oxygen concentration.

A continuous uninterrupted enough supply of oxygen to the brain is crucial to maintain its proper metabolic functions and to avoid tissue damage. However, diseases including cardiorespiratory (heart attack, lung injury, peripheral vascular disease, hypertension, asthma, bronchiectasis, bronchitis etc.), carotid stenosis and cardiovascular disorders (coronary artery disease, heart attack, heart valve disease etc.) results in persistent systemic hypoxia. In hypoxia, the brain is not completely deprived of oxygen, but yet it causes chronic decreased cerebral oxygenation, generation of free radicals, oxidative stress, protein oxidation, altered neurotransmitter synthesis, cellular apoptosis, neuro-degeneration, and memory impairment [45]. In hypoxia, the metabolic support for neuronal signaling gets compromised in many regions of the brain leading to brain injury, neurological and the memory dysfunction [46, 47]. While comparing time-dependent and region-specific changes in the cortex, hippocampus, and striatum upon hypoxia, Maiti *et al.*, [48] found an increase in oxidative stress, free radicals, nitric oxide level, lipid peroxidation, and these authors concluded that the hippocampus is more susceptible to hypoxia than the cortex.

Hypoxia/reoxygenation (H/R) such as induced by ischemia/reperfusion results in neuronal injury mediated by the glutamate/N-methyl-D-aspartate (NMDA)/Ca^++^/nitric oxide (NO) and free radical pathway [49]. The increased neuronal damage with time was noted when primary cultures of rat cortical neurons and glia were exposed to H/R [50]. In hypoxic-ischemic condition, Kim et al [51] noted neuronal death due to the accumulation of Ca^++^ and Na^+^. Variation in oxygen supply to brain tissue in H/R triggers cellular and molecular alterations including protein turnover, protein aggregation, impairment in neural plasticity, perturbed calcium homeostasis, neuronal survival, neuroinflammation etc. which eventually affect brain functions leading to aging and neurological disorders. However, it’s somehow difficult to differentiate normal brain aging and hypoxia-induced aging, because in many cases normal brain aging switches to pathological aging with drastic deterioration in cognitive abilities and motor skills [52].

In hypoxia and under disrupted redox equilibrium due to excessive accumulation or depletion of ROS, the cellular signaling pathways are influenced leading to cellular dysfunction and development of various diseases [53]. Under physiological state, a balance between ROS generation and clearance is regulated by antioxidative defense mechanisms. The major antioxidant enzymes involved are Cu/Zn-superoxide dismutase (Cu/Zn-SOD, SOD1) in the cytosol, manganese superoxide dismutase (Mn-SOD, SOD2) in the mitochondrial matrix, catalase, glutathione peroxidase (GPx), and glutathione reductase (GR). However, when ROS overproduction overcomes intrinsic antioxidant capacity, then the oxidative stress occurs, which damages the biomolecules of cells[54]. The oxidative stress usually results from either excessive ROS production, mitochondrial dysfunction, impaired antioxidant system, or a combination of these factors[54].

Exposing oxygen-sensing pheochromocytoma (PC12) cells, human embryonic kidney cells (HEK-293 in which stably expressed the human cardiac L-type Ca^2+^ channel α1C subunit), the human neuroblastoma (SH-SY5Y), cultures of cortical astrocytes and central neurons to hypoxia (1-2.5% oxygen) for a period of 6-48 h resulted in increased production of amyloidal peptides that affected cell calcium channel currents and impaired calcium signaling [55, 56]. As reviewed by Peers, [56] disrupted oxygen supply to central nervous system promote the formation of ROS, alters mitochondrial energy metabolism, expression of various proteins of calcium homeostasis; causes neuronal death and the onset of dementia. In short, hypoxia induces production of amyloids alters signaling mechanism, causes mitochondrial dysfunction, and neuronal death. On the other hand, these alternations are the root cause of neurodegeneration.

Under the anoxic condition, the brain is completely deprived of oxygen due to sudden cardiac arrest, choking, strangulation, which may lead to major acute damage. Apparently, a few seconds of oxygen deprivation won't cause major damage, but the total circulatory arrest results in loss of ATP production and dysfunction of membrane ATP-dependent Na-K pumps, affect the release of glutamate that further induces excitotoxic injury through NMDA receptors [57, 58]. Activation of the NMDA receptor elevates intracellular calcium, which further increases the free radical level [59, 60]. The elevated level of free radicals is known to cause more damage through lipid peroxidation, protein oxidation, and DNA fragmentation, all of which contribute to cell death [61]. The documented literature clearly indicates the deleterious effects of hypoxia in brain damage, signaling, cellular and metabolic processes. Strokes including both ischemic and hemorrhagic, deprive oxygen in the brain and lead to brain cells death. Desmond et al., [62] found the 4-fold increased risk of incident dementia among ischemic stroke patients who were initially non-demented relative to clinically stroke-free elderly control subjects.

Oxygen is the ultimate electron acceptor in the electron transport chain [48]. The oxygen level in cells remains low in hypoxic conditions, which leads accumulation of electrons. These accumulated electrons attack the ground state of available oxygen to form superoxide anion (O_2_^•^) and chain reaction, leading to the formation of H_2_O_2_ and hydroxyl radicals (OH^• -^). Importantly, hypoxia-induced oxidative stress not only impairs mitochondrial function, neuronal damage/apoptosis through the nitric oxide synthase (NOS) pathway, [63, 64]; but also causes damage to lipids, proteins, and DNA [65]. According to recent literature, cerebral hypoxia, ROS, mitochondrial dysfunction associated with accumulation of amyloid plaque, development of intra-neuronal neurofibrillary tangles, hyperphosphorylated *tau*, loss of synaptic integrity and neuronal death in the cortical region of brain are the main risk factors and causative agents of dementia [66, 67].

Mitochondria are important regulators of longevity, and a direct correlation exists between mitochondrial ROS production and lifespan [68, 69]. Mitochondrial DNA (mtDNA) is a major target for age-associated mutations due to the oxidative environment in mitochondria and lack of efficient histone protective mechanism [6]. The accumulated oxidative stress and ROS lead to oxidation of macromolecules. These oxidized macromolecules are no longer degraded or repaired due to defects in proteostasis [14, 70]. Under hypoxic conditions, protein oxidation and aggregation of lipofuscin in neurons were noted [71]. These events are a hallmark of aging [72]. Hypoxia induces ROS and protein modifications through RNS, primary radical species (•OH, O2•-, CO2•-, NO•), nonradical species (H2O2, HOCl, O3, ONO2-, ONOCO2-, CO, N2O2, NO2, 1O2), and free radicals (•C, RS•, RSO•, RSOO•, RSSR•-, R•, RO•, ROO•)[73]. In addition, proteins can also be modified by highly reactive aldehydes and ketones produced during ROS-mediated oxidation of lipids[74]. Hypoxia alters mitochondrial biogenesis and dynamics[76], and thus leads to mitochondrial dysfunction which is one of the key hallmarks of aging process and numerous age-related pathologies.[75] In summary, technological advances including proteomics, genomics, lipidomics, glycomics, transcriptomics etc. suggest that hypoxia alters mitochondrial biogenesis [76], causes DPMs [77], which leads to aging and age-related neurodegenerative diseases. It’s clear that mitochondria retain a central role in complex balance of cellular processes that contribute to aging, age-associated disorder. The key challenge remains to clinically translate this knowledge into model system and develop therapeutics.

### 2.2. Protein aggregation in hypoxia, aging and neurodegenerative disorders

Amyloidosis is a hallmark of neurodegenerative diseases where different amyloidogenic species cause a variety of neuropathic diseases such as Alzheimer’s disease (AD), Parkinson’s disease (PD), Huntington disease (HD), Prion diseases including Creutzfeldt-Jakob disease, Lewy body disease, Amyotrophic Lateral Sclerosis (ALS) etc. [78]. The protein aggregates are often correlated with diseases, e.g. AD is characterized by aggregates of the tau protein and β-amyloid, PD by alpha-synuclein, and HD by aggregates of the huntingtin protein (Htt). On the contrary, the protein aggregates are not only pathogenic but also exert a protective effect on the stressed striatal neurons [79, 80]. According to Leitman et al [79], the process of forming an aggregate is protective, isolating and segregating the problematic proteins. Arrasate et al [81] found that the inclusion body (IB) formation reduced intracellular level of diffuse protein huntingtin (Htt) and prolonged survival which suggest IB formation protect neurons by decreasing the levels of toxic diffuse forms of Htt. Similar findings of neuroprotective role of aggregated protein were noted in a mouse model of spinocerebellar ataxia in which the polyglutamine-rich forms of the ataxin-1 protein are expressed [82].

Proteins need to be folded into their stable three-dimensional structure for being functional. However, due to the folding errors, mutations, DPMs, failure of the proteostasis network, pathological conditions, and unfavorable conditions like elevated temperature, extreme pH, high pressure and agitation, protein get unfolded/misfolded and aggregation. Aberrant protein aggregation remains a common feature of neurodegenerative diseases, where mis-assembly of Aβ1-42 has been linked to AD. The relative increases in Aβ1-42 levels promote aggregation of Aβ into toxic species. However, aggregation-mediated Aβ1-42 toxicity was reduced in *Caenorhabiditis elegans* when aging was slowed by decreased insulin/insulin growth factor-1-like signaling pathway (IIS) [83, 84]. Thus, modulation of IIS pathway could be a promising approach for the development of AD therapy. It’s important to note that in the nematode *C. elegans*, flies, mice and in human, IIS pathway regulates stress resistance, aging and determines the lifespan [83, 85].

Using *C. elegans* aging model, Kaufman et al., [86] found an aggregation of mitochondrial proteins and failure of mitochondrial protein homeostasis as a characteristic feature of hypoxia, while mitochondrial dysfunction and decreased protein turnover is linked to aging [87]. Recently, Adav et al. [88] evaluated the quantitative profile of Alzheimer disease (AD) brain mitochondrial proteins using both isobaric tags for relative and absolute quantitation (iTRAQ) and label-free quantitative technique and found altered mitochondrial proteins. These authors found destabilization of the junction between the membrane and matrix arm of mitochondria in AD. As evaluated by Kaufman and Crowder, [89] an increase in the detergent-soluble proteins, which are believed to be misfolded and aggregated, were the consequences of the hypoxic conditions. Thus, protein aggregation is common feature of aging and hypoxia and neurodegenerative diseases.

During hypoxia and aging, proteostasis pathway is compromised due to protein aggregation. The proteins of these aggregates have been found associated with neurodegenerative diseases, which indicates that the protein aggregation is an inherent part of aging and neurodegenerative diseases and consequences of hypoxia [90, 91]. Not only aggregation of protein but the protein or peptide-based drug (i.e. small peptides with 7, 6, 5, and 4 residues) is of significant concern [92]. In protein drugs, domain swapping technique is adopted to avoid aggregation, but swapping an aggregation-prone segment from amyloidogenic protein to a non-amyloidogenic homolog triggers amyloid formation [93]. Thus, the short aggregation-prone region of protein/peptide sequence and structural specificity can induce protein aggregation. However, the exact cause of protein aggregation, and the mechanistic link between the protein and tissue degeneration are not yet fully understood.

The protein misfolding and aggregation can induce cellular dysfunctions, cell death, and organelle failure that are the major pathological findings in post-ischemic brain, where impaired autophagy was thought to be the main cause of abnormal proteostasis and protein aggregation [94]. The most prominent structural motif of the functional protein in its native conformation is an alpha helix[95]. The beta sheet conformation also exists in many functional native proteins, but the transition from alpha-helix to beta-sheet is the main feature of amyloids and protein aggregation [95]. In physiological flow conditions, the shear flow induces protein aggregation and amyloid formation [96]. The loosely packed proteins in unfolded state expose their hydrophobic core which may interact with the cellular environment and undergo self-aggregation, while partially folded proteins act as precursors in the protein aggregation process [97].

While investigating the main cause of protein aggregation, the physicochemical analysis revealed that these aggregates differ in size, their proteomic composition and cellular location [98, 99]. Age-related and oxidative stress-induced protein aggregates were having compact conformation and protein carbonylation was a cause of compact aggregation [100]. Based on the solubility, aggregates were classified into soluble and insoluble, where soluble form can be more easily unfolded, while insoluble aggregates accumulate and impair cellular function [101]. When the proteomic composition of soluble and insoluble aggregates of human brain tissue was profiled, it revealed proteins like S100A9, ferritin, hemoglobin subunits, S100-A8, S100-B, collagens, mitochondrial creatine kinase (U-type), β-tubulin and laminin exclusively in compact aggregates and most importantly they were deamidated [23]. The site-specific deamidation revealed deamidation of more than one residue of S100A9, which may introduce a negative charge altering Ca^++^ binding capability and enhancing the capacity of the protein to form pathological aggregates in the brain. Using different aging models including *C. elegans*, murine bone marrow, and spleen cells, DPMs were found as a cause of protein aggregation [90, 91]. In conclusion, recent literature suggests that the ROS generated during hypoxia and aging, increases non-enzymatic DPMs such as carbonylation, oxidation, glycation, deamidation, citrullination, and lipoxidation. These protein modifications alter the protein charge and hydrophobicity, cause protein misfolding and aggregation, and their deposition in brain tissues could be key features of degenerative disease and aging. Despite plentiful evidences of the formation of aggregates and their effects, the exact mechanism(s) of protein aggregation and/or triggering nuclei remains unclear.

### 2.3. Hypoxia-induced DPMs and its role in aging and neurodegeneration

Hypoxia is known to induce broad changes in gene expression. In addition to affecting gene expression, hypoxia also alters the functions of proteins via posttranslational protein modifications. The clinical and experimental studies concluded that cerebrovascular disease and hypoxic-ischemic brain injury are the primary causes of cognitive impairment and dementia [29]. The progressive cycle of hypoxic-ischemic brain injury induces protein misfolding, aggregation and DPMs, leading to cognitive decline and dementia[15]. Further, the changes and posttranslational modification of proteins in response to chronic sustained and intermittent forms of hypoxia have been reviewed by Kumar and Klein [102]. Zanelli et al [103] tested the impact of hypoxia-induced protein nitration on the distribution of nitrotyrosine-like immunoreactivity in the hippocampus of the guinea pig fetus. Hypoxia-induced modifications were documented and reviewed including Slug SUMOylation [104], acetylation [105], glycosylation[106]. Apart from these protein modifications, this review article focuses on hypoxia-induced non-enzymatic post-translational degenerative protein modifications.

#### 2.3.1. Protein Carbonylation

At physiological concentration, ROS is a cellular requirement since it is involved in signaling pathways and in the regulation of numerous cellular activities like cytokine secretion, growth, differentiation and gene expressions, and defense [107]. A cellular balance between ROS production and clearance exits where antioxidant enzymes like Cu/Zn-superoxide dismutase (Cu/Zn-SOD, SOD1), manganese superoxide dismutase (Mn-SOD, SOD2), catalase, glutathione peroxidase (GPx), and glutathione reductase (GR) play a major role[54]. However, when ROS overproduction overcomes intrinsic antioxidant capacity, then the oxidative stress occurs. Thus, beyond the physiological concentration, ROS and secondary by-products of oxidative stress impose detrimental biological damage through protein oxidation. Cysteine and methionine are particularly prone to oxidative attack by ROS. The direct oxidative attack on lysine, arginine, proline or threonine leads to the formation of 2-pyrrolidone from proline, glutamic semialdehyde from arginine and proline, α-aminoadipic semialdehyde from lysine, and 2-amino-3-ketobutyric acid from threonine residues [108, 109]. The ketone carbonyls were also observed from histidine, proline, threonine, and tryptophan residue modification. The secondary reaction of cysteine, histidine, or lysine residues with reactive carbonyl compounds also leads to the formation of protein carbonyl derivatives, aldehydes, and ketones. Aldehydes produced from hydroperoxidation of lipid undergo Schiff-base formation with lysine residues through Michael addition and produces a lipid acyl group containing a free carbonyl that is capable of the secondary Schiff-base formation with an adjacent amine or cyclization. The degradation products of lipid oxidation can also bind mostly to cysteine, lysine, and histidine through Michael addition or Schiff base formation [110, 111].

The accumulation of carbonylated proteins with aging also corresponds to the alterations in hypoxic conditions, but the mechanism that causes accumulation of these proteins, though interesting, but not established. As reviewed by Solaini et al [112], hypoxia modulates mitochondrial oxidative metabolism. Upon exposure of yeast cells to hypoxia, abnormal protein carbonylation and protein tyrosine nitration were noted [113]. An elevated level of protein oxidation has been noted in hypoxic chronic obstructive pulmonary disease (COPD) patients compared with COPD patients who do not exhibit hypoxia [114]. Constant accumulation of oxidized proteins also takes place during aging and the possible causes of accumulation could be i) an increase in the rate of oxidizing species, ii) decrease in scavenging molecules, iii) impaired repair mechanism or iv) decreased clearance of oxidized proteins.

The clearance of oxidized protein is carried out by the proteasomal system. The partial inactivation of the proteasomal system results in the failure of oxidatively damaged protein repair or clearance of oxidized proteins during aging [115]. In mammalian cells, oxidized proteins are removed by the 20S proteasome [116]. The proteasome activity was inhibited or decreased in the episodes of repetitive and intermittent hypoxia in Sprague-Dawley rat brain [117], cerebral ischemia-reperfusion [118] and in aging [119]. In addition to aging, there are several human diseases associated with carbonylated proteins such as AD, amyotrophic lateral sclerosis (ALS), acute respiratory distress syndrome, cataractogenesis, chronic lung disease, dementia with Lewy bodies, diabetes, ischemia-reperfusion, pre-eclampsia [120-123]. Thus, hypoxia induces ROS that leads to protein oxidation causing numerous diseases including neurodegenerative diseases and its shared feature of aging.

To evaluate the correlation between accumulated carbonylated proteins with age, Aguilaniu and colleagues [124] successfully retained higher carbonylated proteins in mother cells compared to daughter cells in the yeast model and confirmed that the accumulation of carbonylated proteins is a characteristic of aging processes. These authors successfully maintained carbonylated protein content in *Saccharomyces cerevisiae* but having such experimental studies in a higher organism such as an animal species is required. The support for the oxidative-stress hypothesis of aging, which postulates that aging is associated with the molecular damage caused by ROS came from the studies by Sohal et al., [125] who found a higher rate of ROS in the mitochondria of older rodents and flies than younger animals.

**Figure 3.**
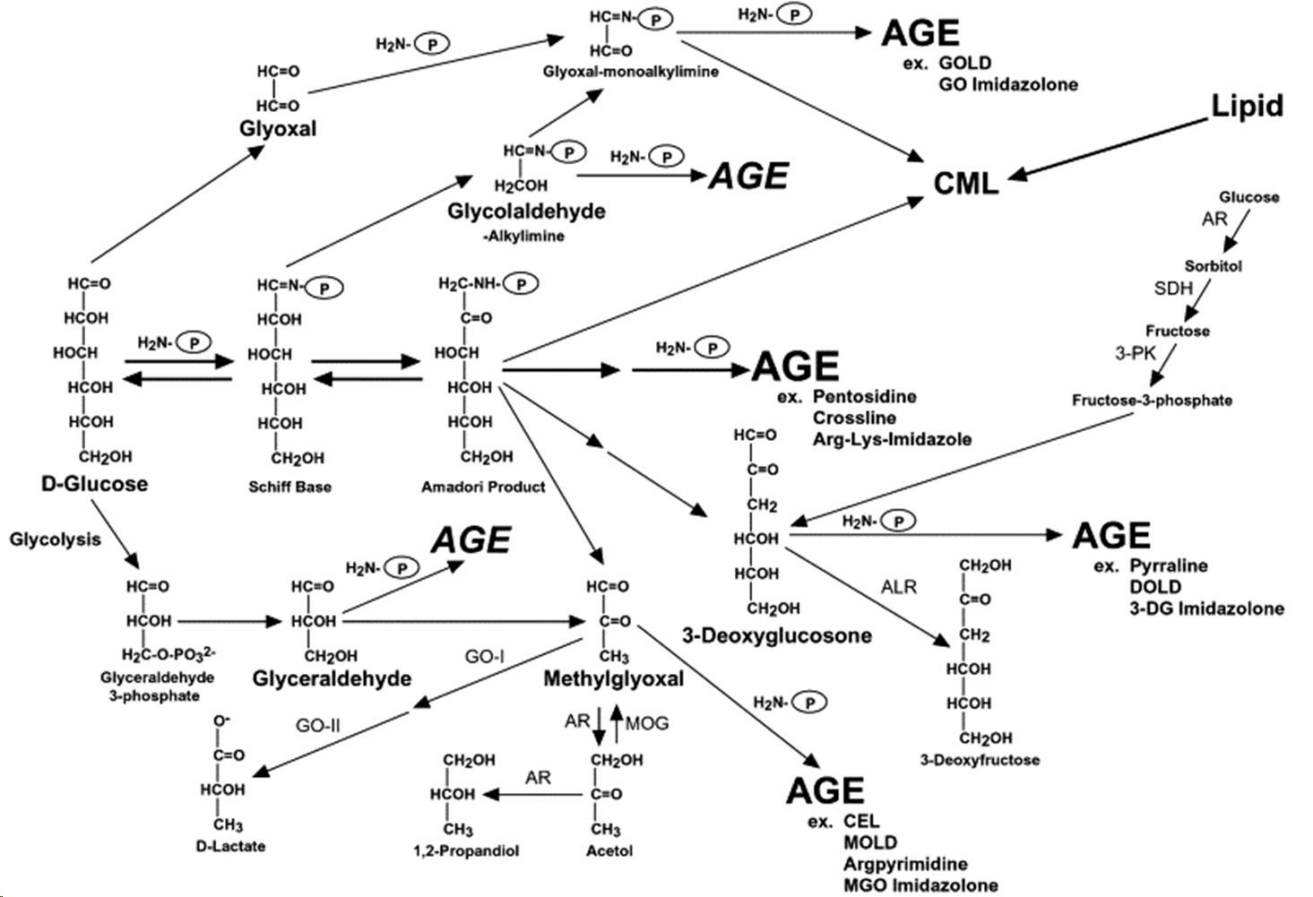
Possible routes of AGE formation (Adopted from Kikuchi et al[208]). AGEs get generated through decomposition of Amadori products, from glycolysis intermediates like glyceraldehyde, a Schiff base fragmentation product such as glycolaldehyde, fragmentation of Amadori products including methylglyoxal and 3-deoxyglucosone, and the autoxidation of glucose to glyoxal. (GO imidazolone, glyoxal imidazolone; MGO imidazolone, methylglyoxal imidazolone).

#### 2.3.2. Protein Glycation

Protein glycosylation is an enzyme-catalysed process that attaches glycans to proteins, lipids, and other organic molecules in a site-specific manner. While, protein glycation is a non-enzymatic reaction between reducing sugar and the free amino group of a target protein. Protein glycation is a consequence of the elevated production of dicarbonyl species such as glyoxal, methylglyoxal, and 3-deoxyglucosone that non-enzymatically react with protein amino groups under conditions of hyperglycemia and oxidative stress. French scientist Louis Camille Maillard, observed a browning reaction when glycine heated with glucose, hence it is called Maillard reactions. It is not a single reaction, but proceed by a series of reactions [126] where the process begins with the reaction of the carbonyl group of glucose with the amino group of a protein to form a Schiff base, followed by conversion into stable Amadori product, which gets further modified to form advanced glycation end-products (AGEs). AGEs formation occurs through decomposition of Amadori products, glycolysis, Schiff base fragmentation product and many more routes as depicted in Fig. 3. Glycation site includes amino groups of protein, particularly, lysines, arginines and N-terminus of amino acids. In addition to protein glycation, DNA glycation also produces AGE, which acts as a potential carcinogen [127]. AGEs are also formed when food is processed at elevated temperatures, and thus, deep-fried, roasted, grilled food act as a source of AGEs; but their importance was largely ignored due to their poor absorption [128]. In mice model, consumption of AGE-rich diets has been linked to conditions such as atherosclerosis and kidney disease[129]. The food-derived AGEs induce protein cross-linking and intracellular oxidant stress similar to their endogenous counterparts [130]. The restriction of AGE-containing diet reduces oxidative stress, AGE accumulation and tissue damage resulting in extension of lifespan in mice [131]. The findings from animal and human studies suggest that chronic diseases and aging can be delayed by avoiding dietary AGEs. In other words, restriction of AGE-rich diet could be a novel therapeutic target to prevent age associated disorders.

AGEs induce damage to cells and extracellular matrix (ECM), thereby contributing to aging and age-related diseases. The buildup and accumulation of AGEs alters the structure and function of proteins, thus affecting several of the hallmarks of aging and responsible for the development of many age-related morbidities [132]. The mechanisms involved are: (i) accumulation of AGEs within the ECM, cross-linking between AGEs and ECM triggering a decrease in tissue elasticity, (ii) modification of proteins, which further results in a loss of the original cellular function, and (iii) interaction of AGEs with receptor for advanced glycation end-products (RAGE), which leads to activation of inflammatory signaling pathways, ROS generation, and apoptosis [133, 134]. The connection between aging, age-related disorders and AGEs remain difficult to unravel due to 1) wide-diverse sources for AGEs, 2) difficulties in detecting accumulation of AGEs, 3) technical limitation in detection and quantitation of AGEs, 4) a lack of models that reiterate the pathologies resulting from the accumulation of AGEs [132]. Moreover, there is no enzyme to remove glycated products from the human body. However, AGEs studies in short lifespans model organisms may help to find answers to their mechanistic role in aging.

Hypoxia condition escalates inside a solid tumor mass due to insufficient oxygen supply. Hypoxia-driven AGE accumulation and RAGE activation is well documented in published literature [135]. Hypoxic conditions elevates expression of RAGE [136] and contribute to protein modifications and, thus influence aging and neurodegeneration processes. Its well-known fact that the cells of hypoxic tumor adopt anaerobic glycolytic process instead of mitochondrial aerobic respiration and these hypoxic cells induce accumulation of di-carbonyls, a precursor of AGE [137]. The methylglyoxal (MG), an intermediate compound generated during glycolysis also act as a precursor molecule for AGE[138]. According to Chang et al [139], a rapid generation of AGE after hypoxia act as a precursor in endothelial cells that further activates RAGE-mediated signaling. These oxygen deficient cells actively participate in tumor growth and metastasis through activating several signaling events[140]. The contribution of AGE in enhancing proliferation of breast cancer progression[141], a key role in prostate cancer[142] has been shown. The mechanism of hypoxia-driven glycation and role of protein glycation in various cancers have been reviewed [143]. Detailed understanding of the AGE mediated cancer onset could open avenues in cancer therapeutics.

Apart from cancer, the roles of AGEs have been linked to various diseases including diabetes, cardio-vascular disease and neurodegenerative disorders [144]. RAGE-AGE interaction mediates myocardial injury after ischemia attack[145]. The hyperglycemia-induced AGE activation followed by retinal neovascularization was studied by Shin *et al.* [146]. Glycation process is a toxic cascade reaction in which different oxidative products with high oxidative potential than the parent compound are produced, and their quantity determines its destructiveness [147]. In fact, the toxicity of glycation results in loss of protein function through cross-linking, aggregation and deposition, and production of reactive species, which are an analogy of hypoxia.

#### 2.3.3. Protein Carbamylation

Carbamylation is an irreversible, non-enzymatic spontaneous reaction of primary amino groups or a free sulfhydryl group of proteins with an isocyanate as reported in the 1960s by Stark et al., [148]. The spontaneous decomposition of urea into ammonium and cyanate generate isocyanic acid, which is a reactive species. The reactive cyanate generated through thiocyanate metabolism and neutrophil-derived myeloperoxidase catalyzes the oxidation of thiocyanate at sites of inflammation and atherosclerotic plaque. Urea is present abundantly in the human body and can decompose spontaneously forming cyanic acid and cyanate, where cyanic acid is in rapid equilibrium with isocyanic acid [148]. When carbamylation occurs on lysine residue then it generates ε-carbamyl-lysine, also called as “homocitrulline” and it remains challenging to differentiate it from citrulline since just with one additional methylene group homocitrulline residue generated by carbamylation becomes structurally similar to the citrulline residue formed by peptidyl arginine deiminase activity [149]. Again, a mass shift of +43 Da (carbamylation) needs to be distinguished from +42 Da (trimethylation or acetylation) to avoid artifactual identification of specific modification. Carbamylation can also occur at the guanidine moiety of arginine and a reduced thiol of cysteine.

Biomolecules such as albumin, low-density lipoprotein (LDL), collagen, and many more, undergo carbamylation in both pathological and physiological conditions that alters these biomolecules structurally and functionally. The accumulation of carbamylated proteins is considered as a hallmark of aging. The carbamylation of crystallin is well studied [150]. The carbamylated erythropoietin (CEPO) is attracting widespread interest due to its neuroprotective effects without erythropoiesis [151]. While other carbamylated proteins namely, carbamylated-haemoglobin and carbamylated-LDL have been implicated in hypoxia and atherosclerosis, respectively [152]. The carbamylation of the erythropoietin (EPO) causes a drastic decrease in its signaling ability and loss in erythropoietic activity. The carbamylation of the erythropoietin is linked to hypoxia in patients with end-stage renal disease [153]. It’s important to mention that in hypoxia, hypoxia-inducible factor 1α (HIF-1α) activates erythropoietin. The distinct receptors for EPO and carbamylated EPO exist. According to Brines et al [154] homodimeric EPO receptor binds only to EPO and regulates its erythropoietic function, while the heterodimeric receptor for carbamylated EPO regulates tissue protection. Thus, carbamylation of EPO results in decreased erythropoiesis and further hypoxia due to the altered synthesis of RBCs and HBs.

#### 2.3.4. Protein deamidation

Deamidation is a non-enzymatic post-translational modification in which amide functional group in the side chain of amino acid asparagine (Asn) is converted into an aspartic acid or iso-aspartic acid, and glutamine (Gln) into glutamic acid. At neutral pH, deamidation introduces a negative charge at the reaction site and lead to biological and structural alterations in peptide and protein. At physiological pH, deamidation is a two-step processes wherein a first step-the peptide bond nitrogen of the N+1 amino acid attacks the carbonyl carbon of the asparagine or aspartate side chain leading to the formation of a five-membered ring structure named as a succinimide or cyclic imide; and in a second step-the succinimide is rapidly hydrolyzed at either the alpha or beta carbonyl group to yield iso-aspartate (beta-aspartate) and aspartate in a ratio of approximately 3:1 [17, 155]. At low pH i.e. pH≤2, direct hydrolysis of the side chain amide generates aspartate as a sole product. At alkaline pH, an elevated rate of succinimide formation has been documented [156] and presumed that the elevated rate is due to greater deprotonation of the peptide bond nitrogen.

Under physiological conditions, L-aspartyl (L-Asp) and L-asparaginyl residues in proteins undergo spontaneously nonenzymatic deamidation leading to the formation of an abnormal isoaspartyl residue. The enzyme protein-L-isoaspartate (D-aspartate) O-methyltransferase (PIMT) recognizes and repairs the abnormal L-isoaspartyl residues in proteins. Since PIMT is an enzyme with methyltransferase activity, Yan et al [157] believe the possibility of regulation of mammalian sterile 20-like kinase (Mst1) activity by PIMT through altering either deamidation or methylation of Mst1. Mst1 regulates apoptosis and tumor suppression in mammals and play a key role in heart disease since its activation causes cardiomyocyte apoptosis and dilated cardiomyopathy. Under hypoxic conditions and UV radiations, Mst1 increases protein deamidation [158]. Thus, it will be fascinating to investigate the role of deamidated Mst1 and impact of PIMT under hypoxic conditions. The possibility of interaction of PIMT with Mst1 and conformational changes of Mst1 can’t be ignored. These studies will highlight the critical role of deamidated Mst1 and PIMT. Further it will shed light on the impact of conformation changes on the formation of the Mst1/Hippo signalling complex with other proteins, such as Rassf1, hWW45, and Lats, which have been shown to play essential roles in the regulation of cardiomyocyte apoptosis and heart failure [159, 160].

Asparagine deamidation was elevated in hyperoxic RBCs relative to normoxia and such phenomena are described as a function of human aging, and RBC senescence [161]. Hypoxic stress also causes chromatin modification including a pool of histone modifications which plays a role in gene regulatory switches [162]. Recent studies implicated the role of protein deamidation in regulating signal transduction in innate immune responses which were reviewed by Zhao et al., [163].

Despite the accumulating evidences, research on the direct impact of DMPs in disease pathology and progression remain limited and still needs further exploration. Proteomics technology remains a state-of-art technology for quantifying DPMs in order to understand their associated biological functions. However, to study the functional alteration that DMPs causes requires a larger toolbox with combination of MS and other more diverse methodology that can highlight the impact of DPMs and help to go beyond DPMs, protein identification and quantification. Future studies are required to focus on the specific DPMs as a therapeutic target and to develop and validate drug targets and effectiveness.

## 3. Role of degenerative protein modifications in aging and age-associated disorders

More than 40 human neurodegenerative diseases have been characterized by proteins misfolding, aggregation, and deposition, including the aggregation of α-synuclein in PD [164], *tau* or beta-amyloid in AD [165, 166], and huntingtin in HD [167] are the examples. Thus, the etiologies of different neurodegenerative disease differ widely but the pathological signature remains protein aggregation. In neurodegenerative diseases including AD, PD, HD, amyotrophic lateral sclerosis (ALS) and prion diseases, protein aggregation remains as a common feature. It’s been believed that the DMP may facilitate protein aggregation. Oxidative modification of protein α-synuclein via dopamine adducts facilitated protein aggregation [168]. Hence, we reviewed the role of DMPs in aging and age-associated disorders as below

### 3.1. Protein Carbonylation

Several theories of dementia and AD are proposed since they were reported a century ago. The initial hypothesis proposed that ischemic cerebral vascular disease or strokes are the main cause of age-related neurodegenerative diseases [169]. The presence of hyperphosphorylated microtubule-associated *tau* protein, intracellular neurofibrillary tangles and extracellular amyloid deposits derived from amyloid precursor proteins in the cerebral cortex and hippocampus are the major hallmarks of neurodegenerative diseases. The injection of Aβ (1-42) into the nucleus basalis of the rat, showed a congophylic deposit and microglial and astrocyte activation which steered a strong inflammatory reaction and induced nitric oxide synthase expression [170]. Aβ is found to be neurotoxic and its neurotoxicity is mediated through its potential to induce free radical mediated oxidative stress, including protein oxidation and lipid peroxidation [171, 172]. In a well-established HD mouse model (R6/2 transgenic mice), α-enolase, γ-enolase, creatine kinase, aconitase, voltage-dependent anion channel 1, and Hsp90 were identified as the main carbonylated proteins that potentially contribute to the impairment of energy metabolism and the pathogenesis of HD [173]. In human brain striatum, Sorolla et al [174] found increased carbonyl levels of glial fibrillary acidic protein, aconitase, γ-enolase (neuron-specific enolase), and creatine kinase B (the brain-specific isoform) in HD patients. Similarly, oxidative and nitrative protein modifications in PD have been documented [175].

Under insufficient oxygen, brain induces oxidative stress, which is indexed by an elevated level of lipid peroxidation, protein oxidation, and neuronal dysfunction or death [121]. Protein carbonyls are the results of a direct free radical attack on the protein backbone or from the products of glycation, glycoxidation, and lipid peroxidation reactions with proteins [176]. Oxidative protein modifications diminish specific protein functions and causes cell death [176, 177]. Mis-regulation of programmed cell death is implicated in aging and age-related neurodegenerative diseases. Protein oxidation is indexed by the presence of protein carbonyls. Protein carbonylation in neurofibrillary tangles, neuronal cell bodies, dendrites and glial nuclei in hippocampal sections have been linked to AD [178]. Further, protein carbonylation in synaptic and non-synaptic mitochondria in the frontal cortex have also been correlated with the AD [179]. Smith et al [180] noted significantly elevated levels of protein oxidation in AD frontal lobe. While Lyras et al [181] noted increased protein carbonyls in the frontal, temporal and occipital lobes and hippocampus in AD.

Boyd-Kimball and colleague [182] identified significantly oxidized numerous proteins which lost their functions due to conformational changes. An altered structural conformation induced protein aggregation, neurofibrillary tangles pathology, loss of synapse and neuronal communication which are associated with the AD. Restated, protein oxidation plays a central role in aging, in the pathogenesis of age-related neurodegenerative diseases, where the possible link could be hypoxia-induced ROS that causes protein oxidation leading to neurodegenerative diseases. Further, an increase in lipid peroxidation, protein oxidation, and accumulation of oxidized biomolecules in neurons exposed to hypoxia may be interpreted as hypoxia accelerate the aging process. In short, hypoxia-induced protein oxidation causes protein aggregation, alter structural conformation and leads to loss of protein function, which is a common feature of aging and age-related disorders.

### 3.2. Protein Glycation and its role in aging and age-associated diseases

It’s well known that the proteins of the extracellular matrix and vascular basement membrane are long-lived. The stiffness of the tissues which are composed of long-lived proteins including skeletal muscle, tendons, joints, bone, heart, arteries, lung, skin, and lens have been correlated with aging [183]. However, these long-lived proteins are susceptible to glycation [184]. The collagen is the most abundant structural protein in skin, tendon, bone, articular cartilage and vascular system; which provides functional properties to different tissues such as renal basement membrane, cardiovascular and retinal capillaries. Hence, the glycation of fibrous and non-fibrous collagen and their correlation with aging remain the subject of research. The glycation of collagen, cross-link between collagen and AGEs in the aorta, carotid and conduit arteries enhances their stiffness with age [185]. Thus, glycation is directly associated with aging. AGE-modified collagen reduces cell migration by impairing mechanism of cell adhesion and proteolytic degradation of collagen by matrix metalloproteinase, [186] while in another study, AGE-modified collagen impaired the potential of chondrocytes of the articular cartilage [187]. Thus, glycation promote collagen cross-linking, increase in tissue stiffness, reduced skin elasticity, modify matrix proteins, and impair several repair processes contributing to vessel rigidity and hypertension, which are an integral part of the aging process. Thus, AGEs could be good therapeutic target to slow down ageing process.

Protein glycation is associated with several neurodegenerative disorders, including AD, PD and HD. AGEs are reported in senile plaques, NFTs, and cerebral amyloid angiopathy from AD brains [188]. In established yeast, mammalian cell and fly models of HD, protein glycation impairs Htt clearance thereby promoting its intracellular accumulation, enhancing its aggregation and pathogenicity [189]. Glycation exacerbates the accumulation, aggregation and toxicity of Aβ and α-synuclein [190]. The protein like tau, Aβ, α-synuclein, and prions were found to be glycated and the extent of glycation is correlated with the pathologies of the patients [191]. AGEs modification triggers the abnormal deposition and accumulation of these modified proteins, and hence it can be hypothesized that glycation contribute to the development of neurodegenerative disease. Thus, drugs targeting the glycation precursors, or promoting the clearance of glycated proteins may be beneficial for AD, PD and HD patients. The targeted studies on characterization of the molecular mechanisms responsible for AGEs mediated neurotoxicity are required to discover the possible link and novel strategies for the prevention and development of therapeutics to treat neuro-degenerative diseases.

Glycation of DNA alters the structure of DNA macromolecule, and it leads to depurination, strand breaks, and mutations such as insertions, deletions, and transposition [192]. Glycation of DNA gives rise to characteristic nucleotide adduct, some of which were found to increase in oxidative stress. Methylglyoxal (MG) is extremely reactive glycating agent that reacts with free amino group of nucleic acids resulting in the formation of DNA-AGEs. MG reacts with guanine residues in DNA to form a tricyclic compound [193] and its mutagenicity at G:C base pairs has been reported [194]. Again, AGE product, carboxylmethyllysine, accumulates in nuclear proteins like histone causing extensive DNA strand cleavage. Thus, DNA-AGEs leads to the loss of genomic integrity during aging and age-related complications. However, recently Richarme and collogues [195] found that parkinsonism-associated protein DJ-1 and its bacterial homologs Hsp31, YhbO, and YajL could repair methylglyoxal- and glyoxal-glycated nucleotides and nucleic acids.

Receptor for advanced glycation end products (RAGE) when binds to AGE, it initiates a further signaling mechanism [196]. RAGE also acts as a receptor for Damage-Associated Molecular Pattern (DAMP) molecule, high mobility group box 1 (HMGB1), the prototypical DAMP, and S100 proteins [196, 197]. Hypoxic conditions elevates expression of RAGE [136] and contribute to protein modifications and, thus impact aging and neurodegeneration processes. AGE-RAGE complex initiates aberrant signaling pathways initiating the onset of diseases such as diabetes, AD, atherosclerosis, heart failure, and various cancers like oral, breast, gastric, colorectal, pancreatic, intestinal and others as reviewed by Khan *et al.*, [198]. AGE-RAGE complex provokes oxidative stress that further induces proliferative, inflammatory, thrombotic and fibrotic reactions [199]. Its well-known fact that the cells of hypoxic tumor adopt anaerobic glycolytic process instead of mitochondrial aerobic respiration and these hypoxic cells induce accumulation of di-carbonyls, a precursor of AGE [137].

Glycation process is a toxic cascade reaction in which different oxidative products with high oxidative potential than the parent compound are produced, and their quantity determines its destructiveness [147]. In fact, the toxicity of glycation results in loss of protein function through cross-linking, aggregation and deposition, and production of reactive species, which are an analogy of hypoxia. The cross-link between adjacent protein and rearrangement of Amodori product remain the cause of protein aggregation and AGEs production, and the consequence of the accumulation of AGEs is plaques and tangles [200]. Accumulated AGEs have also been discovered in senile plaques, primitive plaques, classic plaques and some glial cells of AD brain [201]. It was found that the expression of amyloid precursor protein was elevated *in vitro* and *in vivo* by AGEs, indicating AGEs plays a role in AD pathogenesis [191, 202]. The dimeric form of Apolipoprotein E (ApoE) binds to AGEs and promote the aggregate formation in AD brain by binding to plaque components [203], which may explain how ApoE contributes to increased risk of the AD. In rats, *tau* protein was glycated at its tubulin binding site [204]. This glycated *tau* protein induced oxidative stress and hyperphosphorylation of *tau*, which further impairs memory *via* RAGE-mediated GSK-3 activation. Thus, AGEs play a major role in AD pathogenesis, amyloid formation, protein aggregation, and participates in NFTs formation. AGEs have been intensively studied in diabetes mellitus [205, 206], cardiac dysfunctions [207], visual disorder, nephropathy, vascular disorders [208] and diabetic atherosclerosis.

**Figure 4.**
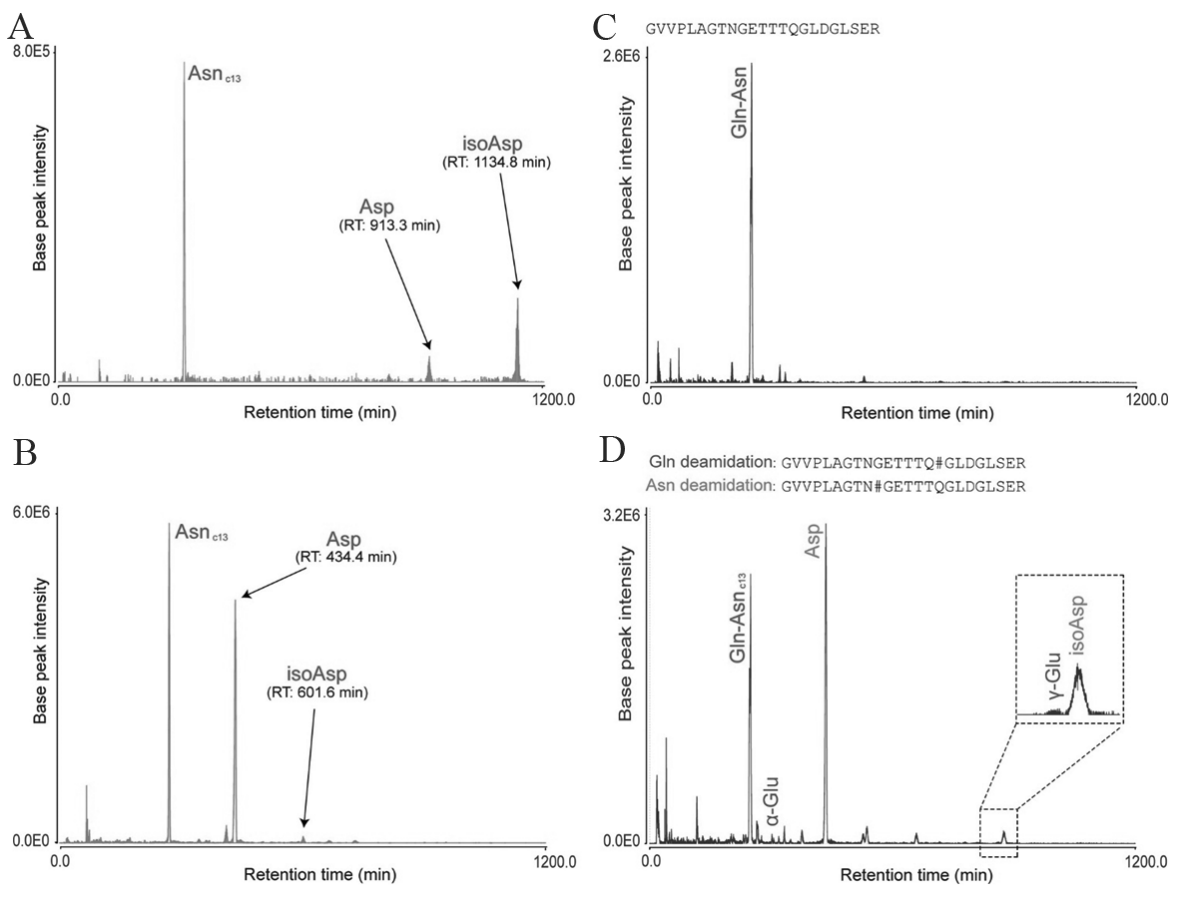
Separation of Asn and Gln deamidation peptides from trypsin-digested human brain tissue using LERLIC-MS/MS. (Adopted from Serra *et al.*[233]) A) Extracted ion chromatograms of peptide N#GFDQCDYGWLSDASVR showing separated triad of Asn deamidated peptides B) Extracted ion chromatograms of VDKGVVPLAGTN#GETTTQGLDGLSER peptide showing separation of carbon-13 peak of nondeamidated peptide (Asn_C13_), Asp (aspartyl isomer) and isoaspartyl isomer (isoAsn), C) Extracted ion chromatograms of GVVPLAGTNGETTTQGLDGLSER nondeamidated peptide, D) Extracted ion chromatograms of two deamidated proteoforms with asn and Gln deamidated residues in peptide GVVPLAGTN#GETTTQGLDGLSER and GVVPLAGTNGETTTQ#GLDGLSER where Gln-Asn_c13_ is a carbon-13 peak of the nondeamidated peptide; α-Glu is α-glutamyl isomer; Asp is Asp aspartyl isomer; γ-Glu is γ-glutamyl isomer; and isoAsp reamin isoaspartyl isomer. #indicates site of modification.

### 3.3 Protein Carbamylation

Carbamylation of the protein causes changes in protein structure and charge, which alter the molecular activity of enzymes, cofactors, hormones, low-density lipoproteins, antibodies, receptors, and transport proteins [209]. Carbamylation induces a conformational change in crystallins, it alters collagen type I structure [210], diminish the activity of insulin and erythropoietin [153], and also induces atherosclerotic plaques formation [211]. The carbamylation of few residues per α-chain of the protein destabilizes its structure leading to decreased thermal stability, fibrillogenesis impairment and altered sensitivity to proteases [210, 212]. Earlier, the quantitative increase in protein carbamylation has been correlated with kidney disease, atherosclerosis, or coronary artery disease [213, 214].

Using murine, bovine, and human species from younger to older ages, an increase in the carbamylated proteins in the skin with time regardless of lifespan have been observed [215]. The type I collagen and elastin of skin was highly carbamylated in older subjects than in younger, and authors projected protein carbamylation as a hallmark of the aging process [215]. Thus, the discovery of carbamylated skin protein has established a link between carbamylation and aging. The carbamylation of brain protein by cyanate [216] caused a decline in learning ability [217], which associate protein carbamylation with neurodegeneration. The carbamylated low-density lipoprotein (cLDL) induced endothelial cell injury, enhanced expression of cell adhesion molecules, and proliferation of vascular smooth muscle cell, which are identical with atherosclerosis [218, 219]. From inflammation to aging, many diseases have been correlated with protein carbamylation and their pathology [215, 220, 221].

### 3.3. Protein deamidation and its role in aging and age-associated diseases

According to comprehensive research by Robinson and collogues [222-226], though, each deamidation site has programmed rate of deamidation, but it also depends on the primary sequence, neighboring amino acids, three-dimensional structure, pH, temperature, ionic strength, and other solution properties. The deamidated Asn and Gln serves as molecular clocks which time biological processes including protein turnover, organismic development, and aging [17, 19, 227, 228]. Deamidation process occurs *in vivo* but it also occurs spontaneously *in vitro* during proteomic sample preparation and introduces artifact. To overcome artefactual identification, Sze and colleagues [229-231] proposed an improved protocol at acidic pH to minimize artefactual non-enzymatic deamidation during sample preparation. Notably, Asn deamidation proceeds via formation of succinimide ring intermediate which gets hydrolyzed to n-Asp and isoAsp [155]. It’s difficult to separate, distinguish and identify n-Asp and isoAsp deamidation products due to their identical mass and charge. The techniques like 2-dimentional reverse phase liquid chromatography coupled with electrostatic repulsion-hydrophilic interaction chromatography (RPLC-ERLIC)-coupled mass spectrometry [232] and long-length electrostatic repulsion-hydrophilic interaction chromatography (LERLIC) tandem mass spectrometry have been optimized for distinguishable separation as well as identification and quantification of deamidation products[233]. The separation of Asn and Gln deamidation peptides by LERIC-MS/MS is shown in Fig 4. An altered brain protein function and aggregation are characteristics of neurodegenerative diseases, however, which specific protein modification recruit plaque formation is not conclusive. But Sze and colleagues proposed that the protein deamidation alters the charge of the protein, influence protein folding, structure, and stability; and initiate protein aggregation in neurodegenerative diseases like dementia [12, 13, 21, 23]. Recently, the critical role of isoDGR motif has also been evaluated by Dutta and Sze et al., [234], the deamidation of extracellular matrix (ECM) proteins in vascular bed and atherosclerotic plaque promotes atherosclerosis by enhancing monocyte-macrophage binding to the ECM proteins in the blood vessel.

The effects of age-dependent protein deamidation were extensively studied in eye lens proteins as a model system due to their exceedingly slow turnover during the patient lifetime. To retain life-long transparency in the absence of protein turnover, eye lens crystalline maintains its longevity by retaining long-term native structure, stability and refolding of non-native proteins by chaperones. However, the disruptive protein modifications, particularly deamidation and accumulation of deamidated proteins remain the main concern. Despite the features that cause age-dependent changes in lens crystallines are still not fully understood, it was found that the aggregate size of crystalline increased with age and was composed of deamidated alpha- and beta- crystalline proteins [235]. Age-related cataract has been characterized by the protein aggregation and DPMs, many reports suggests that lens protein aggregation and deterioration are correlated to protein deamidation and oxidation [236-239]. Age-dependent increase in deamidated histone H1 in the brain of mice and rats over 20-days old to 300-days old [240] also supports the role of deamidation in aging. The proportion of deamidated H1 was 8-fold higher in mice of 10 months when compared with 20 days old animals. It is proposed that the deamidation of linker histone would add negative charge to nucleosome that could influence the binding of DNA, chromatin structure and stability.

## 4. Future perspectives

The main goal of biomedical research is to understand physiological and pathological processes to develop preventive measure and therapeutics to maintain good health throughout life and improve human healthspan. However, maintaining good health become very challenging in the modernized demographic scenario, partly, due to increased oxidative stress that induces various pathologies including ischemic heart disease, cancer, stroke, and diabetes; and neurological disorders. These chronic diseases are the cause of physiological and structural deterioration. With advancing age, several cellular, structural and functional changes occur, where, alterations arisen at pre-mature period are beneficial, but those that occur during the post-maturational period of life are mostly deteriorative, except a few. These deteriorative cellular damages keep accumulating, remain vulnerable to challenges, and lead to a decline in cognitive functions. Therefore, this review attempted to summarize up-to-date information on different aspects of aging, the effects of hypoxia, DPMs during aging and age-associated neurodegenerative diseases. An attempt has been made to find the link between DPMs and aging, as well as the impact of DPMs on age-related pathologies. Hypoxia and disrupted oxygen supply generate oxidative stress, enhance ROS, cellular apoptosis, abnormal mitochondrial metabolism, influence lipid peroxidation, neuro-degeneration and memory impairment, and most importantly increased DPMs. The deposition and accumulation of these modified proteins act as pathogenic factors. These protein damages may not be repairable due to defective repair mechanism. This leads to the aggregation of proteins and their accumulation with age which has been linked to aging and age-associated disorders.

Due to the role and relationship between Aβ in protein aggregation and AD, the so-called "secretases" that produces Aβ have been targeted for development of therapeutics. A new generation of small molecule β-secretase (BACE) inhibitors are being evaluated in clinical trials [241]. The difficulties in direct γ-secretase inhibition, directed the focus towards modifying γ-secretase substrate specificity[242]. Emerging strategies to avoid or clear protein aggregates includes protein reduction using as β-secretase inhibitors/modulators[241], γ-secretase modulators [242], chemotherapy [243], proteasome inhibitors [243], liver transplantation mediated gene therapy [244] etc. Therapeutics used to modulate amyloid deposition and age-associated disorders are reviewed by Yiannopoulou and Papageorgiou [245]. The preclinical studies in mice highlights possible novel immunotherapeutic strategies like DNA epitope vaccine, antibodies against the β-secretase cleavage site of the APP and mucosal vaccination as an effective AD therapy. Removal of hyperphosphorylated tau by immunotherapy inhibits retrograde neurodegeneration [246]. Cholinesterase inhibitors like donepezil (Pfizer, New York, NY, USA), rivastigmine (Novartis, Basel, Switzerland) and galantamine (Janssen, Beerse, Belgium) [247] are being used to delay the decline in cognitive function. Antidepressant drugs like mirtazapine, venlafaxine and duloxetine, combined selective noradrenalin and serotonin inhibitors (SNRIs), and bupropion are being used to treat depression in dementia. However, a further research that focuses on the root cause of the protein aggregation, factors that trigger and sustain aggregate formation is needed.

Neurodegenerative disorders are global public health priorities and require urgent action to address at healthcare level. Elucidating the molecular mechanism and investigating main initiating factors will be a key in developing therapeutic interventions. Administration of N-acetyl cysteine to animals exposed to hypoxia exhibited a decrease in the free radical generation and improvement in memory functions [248], while acetyl-L-Carnitine treatment improved the cognitive capabilities [249]. The implications of such compounds as anti-aging and anti-senescence needs further detailed investigations. AGE-RAGE mediated pathologies are gaining significant importance but the mechanism of different AGE-RAGE mediated signaling pathways need to be explored using proteomic approaches, and we believe such studies will provide new insights into aging and AGE-mediated pathogenesis. Further, such studies in an animal model may discover a direct link between protein modification and their role in aging and age-associated diseases. AGEs are involved in aging, diabetes, cancer, as well as neurodegenerative diseases. Therefore, designing selective inhibitors for AGE could help to treat several diseases. To develop such therapies, it’s essential to correlate Hif1α signaling pathway in hypoxia and other diseases to find out a specific target. The structural modification and aggregation of the protein is a core issue in disease development and need focused detailed research. An advanced mass spectrometry-based proteomic technology can be used to profile global effects since its highly sensitive and powerful tool for global proteome profiling and PTMs/DPMs characterization. Aging and age-associated neurodegenerative diseases are multifactorial, hence adopting unbiased, global, the discovery-driven proteomic approach can emphasize global quantitative proteome with novel protein modifications. Based on this discovery data as a steppingstone, targeted proteomic approach on the DPMs might provide promising insight and assist to elucidate pathologies and develop novel therapies.

## References

[1] Jin K (2010). Modern Biological Theories of Aging. Aging and Disease, 1:72-74. 21132086 PMC2995895

[2] Davidovic M, Sevo G, Svorcan P, Milosevic DP, Despotovic N, Erceg P (2010). Old age as a privilege of the "selfish ones". Aging Dis, 1:139-146. 22396861 PMC3295027

[3] Viña J, Borrás C, Miquel J (2007). Theories of ageing. IUBMB life, 59:249-254. 17505961 10.1080/15216540601178067

[4] Cornelius E (1972). Increased incidence of lymphomas in thymectomized mice--evidence for an immunological theory of aging. Experientia, 28:459. 4556502 10.1007/BF02008340

[5] Harman D (1956). Aging: a theory based on free radical and radiation chemistry. J Gerontol, 11:298-300. 13332224 10.1093/geronj/11.3.298

[6] López-Otín C, Blasco MA, Partridge L, Serrano M, Kroemer G (2013). The Hallmarks of Aging. Cell, 153:1194-1217. 23746838 10.1016/j.cell.2013.05.039 PMC3836174

[7] Liu B, Wang J, Chan KM, Tjia WM, Deng W, Guan X, et al (2005). Genomic instability in laminopathy-based premature aging. Nat Med, 11:780-785. 15980864 10.1038/nm1266

[8] Handy DE, Castro R, Loscalzo J (2011). Epigenetic modifications: basic mechanisms and role in cardiovascular disease. Circulation, 123:2145-2156. 21576679 10.1161/CIRCULATIONAHA.110.956839 PMC3107542

[9] Gallart-Palau X, Serra A, Sze SK (2015). Uncovering Neurodegenerative Protein Modifications via Proteomic Profiling. Int Rev Neurobiol, 121:87-116. 26315763 10.1016/bs.irn.2015.06.002

[10] Gallart-Palau X, Serra A, Lee BST, Guo X, Sze SK (2017). Brain ureido degenerative protein modifications are associated with neuroinflammation and proteinopathy in Alzheimer's disease with cerebrovascular disease. J Neuroinflammation, 14:175. 28865468 10.1186/s12974-017-0946-y PMC5581431

[11] Bourdenx M, Koulakiotis NS, Sanoudou D, Bezard E, Dehay B, Tsarbopoulos A (2017). Protein aggregation and neurodegeneration in prototypical neurodegenerative diseases: Examples of amyloidopathies, tauopathies and synucleinopathies. Prog Neurobiol, 155:171-193. 26209472 10.1016/j.pneurobio.2015.07.003

[12] Hao P, Adav SS, Gallart-Palau X, Sze SK (2017). Recent advances in mass spectrometric analysis of protein deamidation. Mass spectrometry reviews, 36:677-692. 26763661 10.1002/mas.21491

[13] Adav SS, Sze SK (2016). Insight of brain degenerative protein modifications in the pathology of neurodegeneration and dementia by proteomic profiling. Molecular Brain, 9:92. 27809929 10.1186/s13041-016-0272-9 PMC5094070

[14] Wyss-Coray T (2016). Ageing, neurodegeneration and brain rejuvenation. Nature, 539:180-186. 27830812 10.1038/nature20411 PMC5172605

[15] Adav SS, Sze SK 2016 Proteomic Study of Degenerative Protein Modifications in the Molecular Pathology of Neurodegeneration and Dementia. In Update on Dementia: InTech. 10.1186/s13041-016-0272-9 PMC5094070 27809929

[16] Stadtman ER (2001). Protein oxidation in aging and age-related diseases. Ann N Y Acad Sci, 928:22-38. 11795513 10.1111/j.1749-6632.2001.tb05632.x

[17] Robinson AB (1979). Molecular clocks, molecular profiles, and optimum diets: three approaches to the problem of aging. Mech Ageing Dev, 9:225-236. 374893 10.1016/0047-6374(79)90101-5

[18] Robinson NE, Robinson AB (2004). Amide molecular clocks in drosophila proteins: Potential regulators of aging and other processes. Mechanisms of ageing and development, 125:259-267. 15063101 10.1016/j.mad.2004.01.001

[19] Robinson NE, Robinson AB (2001). Molecular clocks. Proceedings of the National Academy of Sciences of the United States of America, 98:944-949. 11158575 10.1073/pnas.98.3.944 PMC14689

[20] Robinson NE, Robinson AB (2001). Deamidation of human proteins. Proceedings of the National Academy of Sciences, 98:12409-12413. 10.1073/pnas.221463198 PMC60067 11606750

[21] Adav SS, Qian J, Ang YL, Kalaria RN, Lai MKP, Chen CP, et al (2014). iTRAQ quantitative clinical proteomics revealed role of Na(+)K(+)-ATPase and its correlation with deamidation in vascular dementia. Journal of Proteome Research, 13:4635-4646. 25152327 10.1021/pr500754j

[22] Gallart-Palau X, Lee BST, Adav SS, Qian J, Serra A, Park JE, et al (2016). Gender differences in white matter pathology and mitochondrial dysfunction in Alzheimer’s disease with cerebrovascular disease. Molecular Brain, 9:9. 26983404 10.1186/s13041-016-0205-7 PMC4794845

[23] Adav SS, Gallart-Palau X, Tan KH, Lim SK, Tam JP, Sze SK (2016). Dementia-linked amyloidosis is associated with brain protein deamidation as revealed by proteomic profiling of human brain tissues. Molecular Brain, 9:353. 10.1186/s13041-016-0200-z PMC4759965 26892330

[24] Sacksteder CA, Qian W-J, Knyushko TV, Wang H, Chin MH, Lacan G, et al (2006). Endogenously nitrated proteins in mouse brain: Links to neurodegenerative disease. Biochemistry, 45:8009-8022. 16800626 10.1021/bi060474w

[25] Ren R-J, Dammer EB, Wang G, Seyfried NT, Levey AI (2014). Proteomics of protein post-translational modifications implicated in neurodegeneration. Translational Neurodegeneration, 3:23. 25671099 10.1186/2047-9158-3-23 PMC4323146

[26] Souza JM, Choi I, Chen Q, Weisse M, Daikhin E, Yudkoff M, et al (2000). Proteolytic degradation of tyrosine nitrated proteins. Archives of biochemistry and biophysics, 380:360-366. 10933892 10.1006/abbi.2000.1940

[27] Desrosiers RR, Fanelus I (2011). Damaged proteins bearing L-isoaspartyl residues and aging: a dynamic equilibrium between generation of isomerized forms and repair by PIMT. Curr Aging Sci, 4:8-18. 21204776

[28] Cataldi A, Di Giulio C (2009). "Oxygen supply" as modulator of aging processes: hypoxia and hyperoxia models for aging studies. Curr Aging Sci, 2:95-102. 20021403 10.2174/1874609810902020095

[29] Kalaria RN, Ihara M (2013). Dementia: Vascular and neurodegenerative pathways—will they meet? Nature Reviews Neurology, 9:487-488. 10.1038/nrneurol.2013.164 23938746

[30] Iadecola C (2013). The pathobiology of vascular dementia. Neuron, 80:844-866. 24267647 10.1016/j.neuron.2013.10.008 PMC3842016

[31] Mukandala G, Tynan R, Lanigan S, O'Connor JJ (2016). The Effects of Hypoxia and Inflammation on Synaptic Signaling in the CNS. Brain sciences, 6:6. 10.3390/brainsci6010006 PMC4810176 26901230

[32] Hoenderdos K, Lodge KM, Hirst RA, Chen C, Palazzo SGC, Emerenciana A, et al (2016). Hypoxia upregulates neutrophil degranulation and potential for tissue injury. Thorax, 71:1030. 27581620 10.1136/thoraxjnl-2015-207604 PMC5099189

[33] McKeown SR (2014). Defining normoxia, physoxia and hypoxia in tumours—implications for treatment response. The British Journal of Radiology, 87:20130676. 24588669 10.1259/bjr.20130676 PMC4064601

[34] Stowe AM, Altay T, Freie AB, Gidday JM (2011). Repetitive hypoxia extends endogenous neurovascular protection for stroke. Ann Neurol, 69:975-985. 21437933 10.1002/ana.22367 PMC3117913

[35] Bigdeli MR (2011). Neuroprotection caused by hyperoxia preconditioning in animal stroke models. ScientificWorldJournal, 11:403-421. 21336456 10.1100/tsw.2011.23 PMC5719998

[36] Gidday JM (2006). Cerebral preconditioning and ischaemic tolerance. Nat Rev Neurosci, 7:437-448. 16715053 10.1038/nrn1927

[37] Carreau A, El Hafny-Rahbi B, Matejuk A, Grillon C, Kieda C (2011). Why is the partial oxygen pressure of human tissues a crucial parameter? Small molecules and hypoxia. J Cell Mol Med, 15:1239-1253. 21251211 10.1111/j.1582-4934.2011.01258.x PMC4373326

[38] Chiang AA (2006). Obstructive sleep apnea and chronic intermittent hypoxia: a review. Chin J Physiol, 49:234-243. 17294831

[39] Kumar GK, Prabhakar NR (2008). POST-TRANSLATIONAL MODIFICATION OF PROTEINS DURING INTERMITTENT HYPOXIA. Respiratory physiology & neurobiology, 164:272-276. 18602876 10.1016/j.resp.2008.05.017 PMC2642904

[40] Barhwal K, Singh SB, Hota SK, Jayalakshmi K, Ilavazhagan G (2007). Acetyl-L-carnitine ameliorates hypobaric hypoxic impairment and spatial memory deficits in rats. Eur J Pharmacol, 570:97-107. 17610872 10.1016/j.ejphar.2007.05.063

[41] Hota SK, Barhwal K, Singh SB, Ilavazhagan G (2007). Differential temporal response of hippocampus, cortex and cerebellum to hypobaric hypoxia: a biochemical approach. Neurochem Int, 51:384-390. 17531352 10.1016/j.neuint.2007.04.003

[42] Gallart-Palau X, Serra A, Hase Y, Tan CF, Chen CP, Kalaria RN, et al (2019). Brain-derived and circulating vesicle profiles indicate neurovascular unit dysfunction in early Alzheimer's disease. Brain Pathol. 10.1111/bpa.12699 PMC8028379 30629763

[43] Shibata M, Yamasaki N, Miyakawa T, Kalaria RN, Fujita Y, Ohtani R, et al (2007). Selective impairment of working memory in a mouse model of chronic cerebral hypoperfusion. Stroke, 38:2826-2832. 17761909 10.1161/STROKEAHA.107.490151

[44] Biswal S, Sharma D, Kumar K, Nag TC, Barhwal K, Hota SK, et al (2016). Global hypoxia induced impairment in learning and spatial memory is associated with precocious hippocampal aging. Neurobiol Learn Mem, 133:157-170. 27246251 10.1016/j.nlm.2016.05.011

[45] Hota KB, Hota SK, Singh SB 2012 Neurodegeneration in Hypoxia: Implications in Aging. In Brain Aging and Therapeutic Interventions. Thakur MK, and Rattan SIS, editors. Dordrecht: Springer Netherlands 177-189.

[46] Riddle DR, Gutierrez G, Zheng D, White LE, Richards A, Purves D (1993). Differential metabolic and electrical activity in the somatic sensory cortex of juvenile and adult rats. J Neurosci, 13:4193-4213. 8410183 10.1523/JNEUROSCI.13-10-04193.1993 PMC6576381

[47] Vannucci RC (1990). Experimental biology of cerebral hypoxia-ischemia: relation to perinatal brain damage. Pediatric Research, 27:317-326. 1971436 10.1203/00006450-199004000-00001

[48] Maiti P, Singh SB, Sharma AK, Muthuraju S, Banerjee PK, Ilavazhagan G (2006). Hypobaric hypoxia induces oxidative stress in rat brain. Neurochemistry International, 49:709-716. 16911847 10.1016/j.neuint.2006.06.002

[49] Zanelli SA, Numagami Y, McGowan JE, Mishra OP, Delivoria-Papadopoulos M (1999). NMDA Receptor-Mediated Calcium Influx in Cerebral Cortical Synaptosomes of the Hypoxic Guinea Pig Fetus. Neurochemical Research, 24:437-446. 10215519 10.1023/a:1020950019986

[50] Wang J-Y, Shum AYC, Wang J-Y (2002). Hypoxia/reoxygenation induces cell injury via different mechanisms in cultured rat cortical neurons and glial cells. Neuroscience Letters, 322:187-191. 11897169 10.1016/s0304-3940(02)00102-7

[51] Won SJ, Kim DY, Gwag BJ (2002). Cellular and molecular pathways of ischemic neuronal death. J Biochem Mol Biol, 35:67-86. 16248972 10.5483/bmbrep.2002.35.1.067

[52] Thakur MK, Konar A, Gautam A 2012 Brain aging: a critical reappraisal. In Brain Aging and Therapeutic Interventions: Springer 1-18.

[53] Zhang J, Wang X, Vikash V, Ye Q, Wu D, Liu Y, et al (2016). ROS and ROS-Mediated Cellular Signaling. Oxidative Medicine and Cellular Longevity, 2016:4350965. 10.1155/2016/4350965 PMC4779832 26998193

[54] Nita M, Grzybowski A (2016). The Role of the Reactive Oxygen Species and Oxidative Stress in the Pathomechanism of the Age-Related Ocular Diseases and Other Pathologies of the Anterior and Posterior Eye Segments in Adults. Oxidative Medicine and Cellular Longevity, 2016:23. 10.1155/2016/3164734 PMC4736974 26881021

[55] Peers C, Smith IF, Boyle JP, Pearson HA (2004). Remodelling of Ca2+ homeostasis in type I cortical astrocytes by hypoxia: evidence for association with Alzheimer's disease. Biol Chem, 385:285-289. 15134342 10.1515/BC.2004.023

[56] Peers C, Pearson HA, Boyle JP (2007). Hypoxia and Alzheimer's disease. Essays Biochem, 43:153-164. 17705799 10.1042/BSE0430153

[57] Vaagenes P, Ginsberg M, Ebmeyer U, Ernster L, Fischer M, Gisvold SE, et al (1996). Cerebral resuscitation from cardiac arrest: pathophysiologic mechanisms. Crit Care Med, 24:S57-68. 8608707

[58] Lipton SA, Rosenberg PA (1994). Excitatory amino acids as a final common pathway for neurologic disorders. N Engl J Med, 330:613-622. 7905600 10.1056/NEJM199403033300907

[59] Choi DW (1992). Excitotoxic cell death. J Neurobiol, 23:1261-1276. 1361523 10.1002/neu.480230915

[60] Traystman RJ, Kirsch JR, Koehler RC (1991). Oxygen radical mechanisms of brain injury following ischemia and reperfusion. J Appl Physiol (1985), 71:1185-1195. 1757340 10.1152/jappl.1991.71.4.1185

[61] Chan PH (2001). Reactive oxygen radicals in signaling and damage in the ischemic brain. J Cereb Blood Flow Metab, 21:2-14. 11149664 10.1097/00004647-200101000-00002

[62] Desmond DW, Moroney JT, Sano M, Stern Y (2002). Incidence of dementia after ischemic stroke: results of a longitudinal study. Stroke, 33:2254-2260. 12215596 10.1161/01.str.0000028235.91778.95

[63] Ma X, Sun Z, Liu Y, Jia Y, Zhang B, Zhang J (2013). Resveratrol improves cognition and reduces oxidative stress in rats with vascular dementia. Neural regeneration research, 8:2050-2059. 25206513 10.3969/j.issn.1673-5374.2013.22.004 PMC4146064

[64] Zhang X, Wu B, Nie K, Jia Y, Yu J (2014). Effects of acupuncture on declined cerebral blood flow, impaired mitochondrial respiratory function and oxidative stress in multi-infarct dementia rats. Neurochemistry International, 65:23-29. 24361538 10.1016/j.neuint.2013.12.004

[65] Paradies G, Petrosillo G, Paradies V, Ruggiero FM (2011). Mitochondrial dysfunction in brain aging: role of oxidative stress and cardiolipin. Neurochemistry international, 58:447-457. 21215780 10.1016/j.neuint.2010.12.016

[66] Zhang F, Niu L, Li S, Le W (2018). Pathological Impacts of Chronic Hypoxia on Alzheimer's Disease. ACS Chem Neurosci. 10.1021/acschemneuro.8b00442 30412668

[67] Villarreal AE, Barron R, Rao KS, Britton GB (2014). The effects of impaired cerebral circulation on Alzheimer's disease pathology: evidence from animal studies. J Alzheimers Dis, 42:707-722. 24927711 10.3233/JAD-140144

[68] Barros MH, da Cunha FM, Oliveira GA, Tahara EB, Kowaltowski AJ (2010). Yeast as a model to study mitochondrial mechanisms in ageing. Mech Ageing Dev, 131:494-502. 20450928 10.1016/j.mad.2010.04.008

[69] Trifunovic A, Wredenberg A, Falkenberg M, Spelbrink JN, Rovio AT, Bruder CE, et al (2004). Premature ageing in mice expressing defective mitochondrial DNA polymerase. Nature, 429:417-423. 15164064 10.1038/nature02517

[70] Brunk UT, Terman A (2002). The mitochondrial-lysosomal axis theory of aging. The FEBS Journal, 269:1996-2002. 10.1046/j.1432-1033.2002.02869.x 11985575

[71] Hota SK, Hota KB, Prasad D, Ilavazhagan G, Singh SB (2010). Oxidative-stress-induced alterations in Sp factors mediate transcriptional regulation of the NR1 subunit in hippocampus during hypoxia. Free Radic Biol Med, 49:178-191. 20381604 10.1016/j.freeradbiomed.2010.03.027

[72] Jung T, Bader N, Grune T (2007). Lipofuscin: formation, distribution, and metabolic consequences. Ann N Y Acad Sci, 1119:97-111. 18056959 10.1196/annals.1404.008

[73] Debevec T, Millet GP, Pialoux V (2017). Hypoxia-Induced Oxidative Stress Modulation with Physical Activity. Frontiers in physiology, 8:84-84. 28243207 10.3389/fphys.2017.00084 PMC5303750

[74] Grimsrud PA, Xie H, Griffin TJ, Bernlohr DA (2008). Oxidative stress and covalent modification of protein with bioactive aldehydes. The Journal of biological chemistry, 283:21837-21841. 18445586 10.1074/jbc.R700019200 PMC2494933

[75] Srivastava S (2017). The mitochondrial basis of aging and age-related disorders. Genes, 8:398. 10.3390/genes8120398 PMC5748716 29257072

[76] Jain K, Prasad D, Singh SB, Kohli E (2015). Hypobaric Hypoxia Imbalances Mitochondrial Dynamics in Rat Brain Hippocampus. Neurology Research International, 2015:12. 10.1155/2015/742059 PMC4509498 26236504

[77] Gillespie MN, Pastukh V, Ruchko MV (2009). Oxidative DNA modifications in hypoxic signaling. Annals of the New York Academy of Sciences, 1177:140-150. 19845616 10.1111/j.1749-6632.2009.05036.x

[78] Iadanza MG, Jackson MP, Hewitt EW, Ranson NA, Radford SE (2018). A new era for understanding amyloid structures and disease. Nat Rev Mol Cell Biol, 19:755-773. 30237470 10.1038/s41580-018-0060-8 PMC7617691

[79] Leitman J, Ulrich Hartl F, Lederkremer GZ (2013). Soluble forms of polyQ-expanded huntingtin rather than large aggregates cause endoplasmic reticulum stress. Nature Communications, 4:2753. 10.1038/ncomms3753 24217578

[80] Haass C, Selkoe DJ (2007). Soluble protein oligomers in neurodegeneration: lessons from the Alzheimer's amyloid beta-peptide. Nat Rev Mol Cell Biol, 8:101-112. 17245412 10.1038/nrm2101

[81] Arrasate M, Mitra S, Schweitzer ES, Segal MR, Finkbeiner S (2004). Inclusion body formation reduces levels of mutant huntingtin and the risk of neuronal death. Nature, 431:805-810. 15483602 10.1038/nature02998

[82] Cummings CJ, Reinstein E, Sun Y, Antalffy B, Jiang Y, Ciechanover A, et al (1999). Mutation of the E6-AP ubiquitin ligase reduces nuclear inclusion frequency while accelerating polyglutamine-induced pathology in SCA1 mice. Neuron, 24:879-892. 10624951 10.1016/s0896-6273(00)81035-1

[83] Cohen E, Paulsson JF, Blinder P, Burstyn-Cohen T, Du D, Estepa G, et al (2009). Reduced IGF-1 signaling delays age-associated proteotoxicity in mice. Cell, 139:1157-1169. 20005808 10.1016/j.cell.2009.11.014 PMC3017511

[84] Cohen E, Bieschke J, Perciavalle RM, Kelly JW, Dillin A (2006). Opposing Activities Protect Against Age-Onset Proteotoxicity. Science, 313:1604. 16902091 10.1126/science.1124646

[85] Kenyon C (2005). The plasticity of aging: insights from long-lived mutants. Cell, 120:449-460. 15734678 10.1016/j.cell.2005.02.002

[86] Kaufman DM, Wu X, Scott BA, Itani OA, Van Gilst MR, Bruce JE, et al (2017). Ageing and hypoxia cause protein aggregation in mitochondria. Cell Death Differ, 24:1730-1738. 28644434 10.1038/cdd.2017.101 PMC5596417

[87] Dhondt I, Petyuk VA, Bauer S, Brewer HM, Smith RD, Depuydt G, et al (2017). Changes of Protein Turnover in Aging Caenorhabditis elegans. Mol Cell Proteomics, 16:1621-1633. 28679685 10.1074/mcp.RA117.000049 PMC5587862

[88] Adav SS, Park JE, Sze SK (2019). Quantitative profiling brain proteomes revealed mitochondrial dysfunction in Alzheimer’s disease. Molecular Brain, 12:8. 30691479 10.1186/s13041-019-0430-y PMC6350377

[89] Kaufman DM, Crowder CM (2015). Mitochondrial Proteostatic Collapse Leads to Hypoxic Injury. Curr Biol, 25:2171-2176. 26234215 10.1016/j.cub.2015.06.062 PMC4938157

[90] David DC, Ollikainen N, Trinidad JC, Cary MP, Burlingame AL, Kenyon C (2010). Widespread protein aggregation as an inherent part of aging in C. elegans. PLoS Biol, 8:e1000450. 20711477 10.1371/journal.pbio.1000450 PMC2919420

[91] Cannizzo ES, Clement CC, Sahu R, Follo C, Santambrogio L (2011). Oxidative stress, inflamm-aging and immunosenescence. J Proteomics, 74:2313-2323. 21718814 10.1016/j.jprot.2011.06.005

[92] Fang Y, Gao S, Tai D, Middaugh CR, Fang J (2013). Identification of properties important to protein aggregation using feature selection. BMC Bioinformatics, 14:314-314. 24165390 10.1186/1471-2105-14-314 PMC3819749

[93] Galzitskaya OV (2011). Regions which are Responsible for Swapping are also Responsible for Folding and Misfolding. Open Biochem J, 5:27-36. 21769300 10.2174/1874091X01105010027 PMC3134983

[94] Luo T, Park Y, Sun X, Liu C, Hu B (2013). Protein misfolding, aggregation, and autophagy after brain ischemia. Transl Stroke Res, 4:581-588. 24323413 10.1007/s12975-013-0299-5

[95] Pauling L, Corey RB, Branson HR (1951). The structure of proteins: Two hydrogen-bonded helical configurations of the polypeptide chain. Proceedings of the National Academy of Sciences, 37:205-211. 10.1073/pnas.37.4.205 PMC1063337 14816373

[96] Hill EK, Krebs B, Goodall DG, Howlett GJ, Dunstan DE (2006). Shear flow induces amyloid fibril formation. Biomacromolecules, 7:10-13. 16398490 10.1021/bm0505078

[97] Fink AL (1998). Protein aggregation: folding aggregates, inclusion bodies and amyloid. Folding and Design, 3:R9-R23. 9502314 10.1016/S1359-0278(98)00002-9

[98] Tyedmers J, Mogk A, Bukau B (2010). Cellular strategies for controlling protein aggregation. Nat Rev Mol Cell Biol, 11:777-788. 20944667 10.1038/nrm2993

[99] Lelouard H, Gatti E, Cappello F, Gresser O, Camosseto V, Pierre P (2002). Transient aggregation of ubiquitinated proteins during dendritic cell maturation. Nature, 417:177-182. 12000969 10.1038/417177a

[100] Tanase M, Urbanska AM, Zolla V, Clement CC, Huang L, Morozova K, et al (2016). Role of Carbonyl Modifications on Aging-Associated Protein Aggregation. Scientific Reports, 6:19311. 26776680 10.1038/srep19311 PMC4726109

[101] Kopito RR (2000). Aggresomes, inclusion bodies and protein aggregation. Trends Cell Biol, 10:524-530. 11121744 10.1016/s0962-8924(00)01852-3

[102] Kumar GK, Klein JB (2004). Analysis of expression and posttranslational modification of proteins during hypoxia. Journal of Applied Physiology, 96:1178-1186. 14766768 10.1152/japplphysiol.00818.2003

[103] Zanelli S, Spandou E, Mishra OP, Legido A, Delivoria-Papadopoulos M, Katsetos CD (1999). Hypoxia-Induced Nitration of Protein in the Hippocampus of the Guinea Pig Fetus. Pediatric Research, 45:350A.10088653

[104] Hung PF, Hong TM, Chang CC, Hung CL, Hsu YL, Chang YL, et al (2019). Hypoxia-induced Slug SUMOylation enhances lung cancer metastasis. J Exp Clin Cancer Res, 38:5. 30612578 10.1186/s13046-018-0996-8 PMC6322271

[105] Dhillon RS, Richards JG (2018). Hypoxia induces selective modifications to the acetylome in the brain of zebrafish (Danio rerio). Comp Biochem Physiol B Biochem Mol Biol, 224:79-87. 29309913 10.1016/j.cbpb.2017.12.018

[106] Peixoto A, Fernandes E, Gaiteiro C, Lima L, Azevedo R, Soares J, et al (2016). Hypoxia enhances the malignant nature of bladder cancer cells and concomitantly antagonizes protein O-glycosylation extension. Oncotarget, 7:63138-63157. 27542232 10.18632/oncotarget.11257 PMC5325352

[107] Hensley K, Robinson KA, Gabbita SP, Salsman S, Floyd RA (2000). Reactive oxygen species, cell signaling, and cell injury. Free Radical Biology and Medicine, 28:1456-1462. 10927169 10.1016/s0891-5849(00)00252-5

[108] Requena JR, Chao CC, Levine RL, Stadtman ER (2001). Glutamic and aminoadipic semialdehydes are the main carbonyl products of metal-catalyzed oxidation of proteins. Proc Natl Acad Sci U S A, 98:69-74. 11120890 10.1073/pnas.011526698 PMC14546

[109] Dalle-Donne I, Giustarini D, Colombo R, Rossi R, Milzani A (2003). Protein carbonylation in human diseases. Trends in Molecular Medicine, 9:169-176. 12727143 10.1016/s1471-4914(03)00031-5

[110] Isom AL, Barnes S, Wilson L, Kirk M, Coward L, Darley-Usmar V (2004). Modification of Cytochrome c by 4-hydroxy- 2-nonenal: evidence for histidine, lysine, and arginine-aldehyde adducts. J Am Soc Mass Spectrom, 15:1136-1147. 15276160 10.1016/j.jasms.2004.03.013

[111] Magni F, Galbusera C, Tremolada L, Ferrarese C, Kienle MG (2002). Characterisation of adducts of the lipid peroxidation product 4-hydroxy-2-nonenal and amyloid beta-peptides by liquid chromatography/electrospray ionisation mass spectrometry. Rapid Commun Mass Spectrom, 16:1485-1493. 12125026 10.1002/rcm.743

[112] Solaini G, Baracca A, Lenaz G, Sgarbi G (2010). Hypoxia and mitochondrial oxidative metabolism. Biochimica et Biophysica Acta (BBA) - Bioenergetics, 1797:1171-1177. 20153717 10.1016/j.bbabio.2010.02.011

[113] Poyton RO, Ball KA, Castello PR (2009). Mitochondrial generation of free radicals and hypoxic signaling. Trends Endocrinol Metab, 20:332-340. 19733481 10.1016/j.tem.2009.04.001

[114] Koechlin C, Maltais F, Saey D, Michaud A, Leblanc P, Hayot M, et al (2005). Hypoxaemia enhances peripheral muscle oxidative stress in chronic obstructive pulmonary disease. Thorax, 60:834-841. 15964914 10.1136/thx.2004.037531 PMC1747208

[115] Stolzing A, Grune T (2001). The proteasome and its function in the ageing process. Clinical and Experimental Dermatology, 26:566-572. 11696059 10.1046/j.1365-2230.2001.00867.x

[116] Grune T, Reinheckel T, Davies KJ (1997). Degradation of oxidized proteins in mammalian cells. Faseb j, 11:526-534. 9212076

[117] Gozal D, Row BW, Kheirandish L, Liu R, Guo SZ, Qiang F, et al (2003). Increased susceptibility to intermittent hypoxia in aging rats: changes in proteasomal activity, neuronal apoptosis and spatial function. Journal of neurochemistry, 86:1545-1552. 12950463 10.1046/j.1471-4159.2003.01973.x

[118] Wojcik C, Di Napoli M (2004). Ubiquitin-proteasome system and proteasome inhibition: new strategies in stroke therapy. Stroke, 35:1506-1518. 15118168 10.1161/01.STR.0000126891.93919.4e

[119] Keller J, Huang F, Markesbery W (2000). Decreased levels of proteasome activity and proteasome expression in aging spinal cord. Neuroscience, 98:149-156. 10858621 10.1016/s0306-4522(00)00067-1

[120] Castegna A, Aksenov M, Thongboonkerd V, Klein JB, Pierce WM, Booze R, et al (2002). Proteomic identification of oxidatively modified proteins in Alzheimer's disease brain. Part II: dihydropyrimidinase-related protein 2, alpha-enolase and heat shock cognate 71. J Neurochem, 82:1524-1532. 12354300 10.1046/j.1471-4159.2002.01103.x

[121] Butterfield DA, Lauderback CM (2002). Lipid peroxidation and protein oxidation in Alzheimer's disease brain: potential causes and consequences involving amyloid beta-peptide-associated free radical oxidative stress. Free Radic Biol Med, 32:1050-1060. 12031889 10.1016/s0891-5849(02)00794-3

[122] Boscia F, Grattagliano I, Vendemiale G, Micelli-Ferrari T, Altomare E (2000). Protein oxidation and lens opacity in humans. Invest Ophthalmol Vis Sci, 41:2461-2465. 10937554

[123] Telci A, Çakatay U, Salman S, Satman İ, Sivas A (2000). Oxidative protein damage in early stage Type 1 diabetic patients. Diabetes Research and Clinical Practice, 50:213-223. 11106836 10.1016/s0168-8227(00)00197-2

[124] Aguilaniu H, Gustafsson L, Rigoulet M, Nystrom T (2003). Asymmetric inheritance of oxidatively damaged proteins during cytokinesis. Science, 299:1751-1753. 12610228 10.1126/science.1080418

[125] Sohal RS, Agarwal S, Dubey A, Orr WC (1993). Protein oxidative damage is associated with life expectancy of houseflies. Proc Natl Acad Sci U S A, 90:7255-7259. 8346242 10.1073/pnas.90.15.7255 PMC47115

[126] Tessier FJ (2010). The Maillard reaction in the human body. The main discoveries and factors that affect glycation. Pathologie Biologie, 58:214-219. 19896783 10.1016/j.patbio.2009.09.014

[127] Ashraf JM, Shahab U, Tabrez S, Lee EJ, Choi I, Aslam Yusuf M, et al (2016). DNA Glycation from 3-Deoxyglucosone Leads to the Formation of AGEs: Potential Role in Cancer Auto-antibodies. Cell Biochem Biophys, 74:67-77. 26972303 10.1007/s12013-015-0713-6

[128] Ulrich P, Cerami A (2001). Protein glycation, diabetes, and aging. Recent Prog Horm Res, 56:1-21. 11237208 10.1210/rp.56.1.1

[129] Uribarri J, Woodruff S, Goodman S, Cai W, Chen X, Pyzik R, et al (2010). Advanced glycation end products in foods and a practical guide to their reduction in the diet. Journal of the American Dietetic Association, 110:911-916.e912. 20497781 10.1016/j.jada.2010.03.018 PMC3704564

[130] Cai W, Gao QD, Zhu L, Peppa M, He C, Vlassara H (2002). Oxidative stress-inducing carbonyl compounds from common foods: novel mediators of cellular dysfunction. Mol Med, 8:337-346. 12393931 PMC2040002

[131] Cai W, He JC, Zhu L, Chen X, Wallenstein S, Striker GE, et al (2007). Reduced oxidant stress and extended lifespan in mice exposed to a low glycotoxin diet: association with increased AGER1 expression. Am J Pathol, 170:1893-1902. 17525257 10.2353/ajpath.2007.061281 PMC1899464

[132] Chaudhuri J, Bains Y, Guha S, Kahn A, Hall D, Bose N, et al (2018). The Role of Advanced Glycation End Products in Aging and Metabolic Diseases: Bridging Association and Causality. Cell Metabolism, 28:337-352. 30184484 10.1016/j.cmet.2018.08.014 PMC6355252

[133] Kim C-S, Park S, Kim J (2017). The role of glycation in the pathogenesis of aging and its prevention through herbal products and physical exercise. Journal of exercise nutrition & biochemistry, 21:55-61. 29036767 10.20463/jenb.2017.0027 PMC5643203

[134] Schalkwijk CG, Miyata T (2012). Early-and advanced non-enzymatic glycation in diabetic vascular complications: the search for therapeutics. Amino acids, 42:1193-1204. 20960212 10.1007/s00726-010-0779-9 PMC3296013

[135] Gopal P, Gosker HR, Theije CC, Eurlings IM, Sell DR, Monnier VM, et al (2015). Effect of chronic hypoxia on RAGE and its soluble forms in lungs and plasma of mice. Biochim Biophys Acta, 1852:992-1000. 25703138 10.1016/j.bbadis.2015.02.003 PMC5102336

[136] Reynolds PR, Schmitt RE, Kasteler SD, Sturrock A, Sanders K, Bierhaus A, et al (2010). Receptors for advanced glycation end-products targeting protect against hyperoxia-induced lung injury in mice. Am J Respir Cell Mol Biol, 42:545-551. 19541845 10.1165/rcmb.2008-0265OC

[137] Lin JA, Wu CH, Lu CC, Hsia SM, Yen GC (2016). Glycative stress from advanced glycation end products (AGEs) and dicarbonyls: An emerging biological factor in cancer onset and progression. Mol Nutr Food Res, 60:1850-1864. 26774083 10.1002/mnfr.201500759

[138] Allaman I, Bélanger M, Magistretti PJ (2015). Methylglyoxal, the dark side of glycolysis. Frontiers in neuroscience, 9:23. 25709564 10.3389/fnins.2015.00023 PMC4321437

[139] Chang JS, Wendt T, Qu W, Kong L, Zou YS, Schmidt AM, et al (2008). Oxygen deprivation triggers upregulation of early growth response-1 by the receptor for advanced glycation end products. Circulation research, 102:905-913. 18323529 10.1161/CIRCRESAHA.107.165308

[140] Rojas A, González I, Morales E, Pérez-Castro R, Romero J, Figueroa H (2011). Diabetes and cancer: Looking at the multiligand/RAGE axis. World journal of diabetes, 2:108. 21860695 10.4239/wjd.v2.i7.108 PMC3158864

[141] Sharaf H, Matou-Nasri S, Wang Q, Rabhan Z, Al-Eidi H, Al Abdulrahman A, et al (2015). Advanced glycation endproducts increase proliferation, migration and invasion of the breast cancer cell line MDA-MB-231. Biochimica et Biophysica Acta (BBA)-Molecular Basis of Disease, 1852:429-441. 25514746 10.1016/j.bbadis.2014.12.009

[142] Rodriguez-Teja M, Gronau JH, Breit C, Zhang YZ, Minamidate A, Caley MP, et al (2015). AGE-modified basement membrane cooperates with Endo180 to promote epithelial cell invasiveness and decrease prostate cancer survival. The Journal of pathology, 235:581-592. 25408555 10.1002/path.4485

[143] Khan MI, Rath S, Adhami VM, Mukhtar H (2018). Hypoxia driven glycation: Mechanisms and therapeutic opportunities. Seminars in Cancer Biology, 49:75-82. 28546110 10.1016/j.semcancer.2017.05.008 PMC5699980

[144] Ahmad S, Khan MS, Akhter F, Khan MS, Khan A, Ashraf J, et al (2014). Glycoxidation of biological macromolecules: a critical approach to halt the menace of glycation. Glycobiology, 24:979-990. 24946787 10.1093/glycob/cwu057

[145] Bucciarelli LG, Kaneko M, Ananthakrishnan R, Harja E, Lee LK, Hwang YC, et al (2006). Receptor for advanced-glycation end products: key modulator of myocardial ischemic injury. Circulation, 113:1226-1234. 16505177 10.1161/CIRCULATIONAHA.105.575993

[146] Shin ES, Sorenson CM, Sheibani N (2014). Diabetes and retinal vascular dysfunction. Journal of ophthalmic & vision research, 9:362. 25667739 10.4103/2008-322X.143378 PMC4307665

[147] Taghavi F, Habibi-Rezaei M, Amani M, Saboury AA, Moosavi-Movahedi AA (2017). The status of glycation in protein aggregation. Int J Biol Macromol, 100:67-74. 26751401 10.1016/j.ijbiomac.2015.12.085

[148] Stark GR, Stein WH, Moore S (1960). Reactions of the cyanate present in aqueous urea with amino acids and proteins. Journal of Biological Chemistry, 235:3177-3181.

[149] Jaisson S, Pietrement C, Gillery P (2011). Carbamylation-derived products: bioactive compounds and potential biomarkers in chronic renal failure and atherosclerosis. Clin Chem, 57:1499-1505. 21768218 10.1373/clinchem.2011.163188

[150] Verbrugge FH, Tang WW, Hazen SL (2015). Protein carbamylation and cardiovascular disease. Kidney international, 88:474-478. 26061545 10.1038/ki.2015.166 PMC4556561

[151] Ding J, Wang J, Li Q-Y, Yu J-Z, Ma C-G, Wang X, et al (2017). Neuroprotection and CD131/GDNF/AKT pathway of carbamylated erythropoietin in hypoxic neurons. Molecular neurobiology, 54:5051-5060. 27541284 10.1007/s12035-016-0022-0

[152] Badar A, Arif Z, Alam K (2018). Role of carbamylated biomolecules in human diseases. IUBMB life, 70:267-275. 29542227 10.1002/iub.1732

[153] Park KD, Mun KC, Chang EJ, Park SB, Kim HC (2004). Inhibition of erythropoietin activity by cyanate. Scand J Urol Nephrol, 38:69-72. 15204430 10.1080/00365590310006291

[154] Brines M, Grasso G, Fiordaliso F, Sfacteria A, Ghezzi P, Fratelli M, et al (2004). Erythropoietin mediates tissue protection through an erythropoietin and common beta-subunit heteroreceptor. Proc Natl Acad Sci U S A, 101:14907-14912. 15456912 10.1073/pnas.0406491101 PMC522054

[155] Geiger T, Clarke S (1987). Deamidation, isomerization, and racemization at asparaginyl and aspartyl residues in peptides. Succinimide-linked reactions that contribute to protein degradation. J Biol Chem, 262:785-794. 3805008

[156] Song Y, Schowen RL, Borchardt RT, Topp EM (2001). Effect of 'pH' on the rate of asparagine deamidation in polymeric formulations: 'pH'-rate profile. J Pharm Sci, 90:141-156. 11169531 10.1002/1520-6017(200102)90:2<141::aid-jps5>3.0.co;2-y

[157] Yan G, Qin Q, Yi B, Chuprun K, Sun H, Huang S, et al (2013). Protein-L-isoaspartate (D-aspartate) O-methyltransferase protects cardiomyocytes against hypoxia induced apoptosis through inhibiting proapoptotic kinase Mst1. International journal of cardiology, 168:3291-3299. 23647599 10.1016/j.ijcard.2013.04.045 PMC3788851

[158] Mafia K, Gupta R, Kirk M, Wilson L, Srivastava OP, Barnes S (2008). UV-A-induced structural and functional changes in human lens deamidated alphaB-crystallin. Mol Vis, 14:234-248. 18334940 PMC2255029

[159] Matsui Y, Nakano N, Shao D, Gao S, Luo W, Hong C, et al (2008). Lats2 is a negative regulator of myocyte size in the heart. Circ Res, 103:1309-1318. 18927464 10.1161/CIRCRESAHA.108.180042 PMC2775813

[160] Del Re DP, Matsuda T, Zhai P, Gao S, Clark GJ, Van Der Weyden L, et al (2010). Proapoptotic Rassf1A/Mst1 signaling in cardiac fibroblasts is protective against pressure overload in mice. J Clin Invest, 120:3555-3567. 20890045 10.1172/JCI43569 PMC2947240

[161] Reisz JA, Nemkov T, Dzieciatkowska M, Culp-Hill R, Stefanoni D, Hill RC, et al (2018). Methylation of protein aspartates and deamidated asparagines as a function of blood bank storage and oxidative stress in human red blood cells. Transfusion, 58:2978-2991. 30312994 10.1111/trf.14936 PMC6357231

[162] Johnson AB, Denko N, Barton MC (2008). Hypoxia induces a novel signature of chromatin modifications and global repression of transcription. Mutation research, 640:174-179. 18294659 10.1016/j.mrfmmm.2008.01.001 PMC2346607

[163] Zhao J, Li J, Xu S, Feng P (2016). Emerging Roles of Protein Deamidation in Innate Immune Signaling. J Virol, 90:4262-4268. 26889032 10.1128/JVI.01980-15 PMC4836359

[164] Cuervo AM, Stefanis L, Fredenburg R, Lansbury PT, Sulzer D (2004). Impaired degradation of mutant alpha-synuclein by chaperone-mediated autophagy. Science, 305:1292-1295. 15333840 10.1126/science.1101738

[165] Lemere CA, Lopera F, Kosik KS, Lendon CL, Ossa J, Saido TC, et al (1996). The E280A presenilin 1 Alzheimer mutation produces increased A beta 42 deposition and severe cerebellar pathology. Nat Med, 2:1146-1150. 8837617 10.1038/nm1096-1146

[166] Phiel CJ, Wilson CA, Lee VM, Klein PS (2003). GSK-3alpha regulates production of Alzheimer's disease amyloid-beta peptides. Nature, 423:435-439. 12761548 10.1038/nature01640

[167] DiFiglia M, Sapp E, Chase KO, Davies SW, Bates GP, Vonsattel JP, et al (1997). Aggregation of huntingtin in neuronal intranuclear inclusions and dystrophic neurites in brain. Science, 277:1990-1993. 9302293 10.1126/science.277.5334.1990

[168] Conway KA, Rochet J-C, Bieganski RM, Lansbury PT (2001). Kinetic stabilization of the α-synuclein protofibril by a dopamine-α-synuclein adduct. Science, 294:1346-1349. 11701929 10.1126/science.1063522

[169] de la Torre JC (2012). Cardiovascular risk factors promote brain hypoperfusion leading to cognitive decline and dementia. Cardiovasc Psychiatry Neurol, 2012:367516. 10.1155/2012/367516 PMC3518077 23243502

[170] Giovannini MG, Scali C, Prosperi C, Bellucci A, Vannucchi MG, Rosi S, et al (2002). Beta-amyloid-induced inflammation and cholinergic hypofunction in the rat brain in vivo: involvement of the p38MAPK pathway. Neurobiol Dis, 11:257-274. 12505419 10.1006/nbdi.2002.0538

[171] Butterfield DA (2002). Amyloid beta-peptide (1-42)-induced oxidative stress and neurotoxicity: implications for neurodegeneration in Alzheimer's disease brain. A review. Free Radic Res, 36:1307-1313. 12607822 10.1080/1071576021000049890

[172] Butterfield DA, Drake J, Pocernich C, Castegna A (2001). Evidence of oxidative damage in Alzheimer's disease brain: central role for amyloid beta-peptide. Trends Mol Med, 7:548-554. 11733217 10.1016/s1471-4914(01)02173-6

[173] Perluigi M, Poon HF, Maragos W, Pierce WM, Klein JB, Calabrese V, et al (2005). Proteomic analysis of protein expression and oxidative modification in r6/2 transgenic mice: a model of Huntington disease. Molecular & Cellular Proteomics, 4:1849-1861. 15968004 10.1074/mcp.M500090-MCP200

[174] Sorolla MA, Reverter-Branchat G, Tamarit J, Ferrer I, Ros J, Cabiscol E (2008). Proteomic and oxidative stress analysis in human brain samples of Huntington disease. Free Radical Biology and Medicine, 45:667-678. 18588971 10.1016/j.freeradbiomed.2008.05.014

[175] Danielson SR, Andersen JK (2008). Oxidative and nitrative protein modifications in Parkinson's disease. Free radical biology & medicine, 44:1787-1794. 18395015 10.1016/j.freeradbiomed.2008.03.005 PMC2422863

[176] Butterfield DA, Stadtman ER 1997 Protein oxidation processes in aging brain. In Advances in Cell Aging and Gerontology: Elsevier 161-191.

[177] Stadtman ER (1992). Protein oxidation and aging. Science, 257:1220-1224. 1355616 10.1126/science.1355616

[178] Smith MA, Sayre LM, Anderson VE, Harris PL, Beal MF, Kowall N, et al (1998). Cytochemical demonstration of oxidative damage in Alzheimer disease by immunochemical enhancement of the carbonyl reaction with 2,4-dinitrophenylhydrazine. J Histochem Cytochem, 46:731-735. 9603784 10.1177/002215549804600605

[179] Ansari MA, Scheff SW (2010). Oxidative stress in the progression of Alzheimer disease in the frontal cortex. J Neuropathol Exp Neurol, 69:155-167. 20084018 10.1097/NEN.0b013e3181cb5af4 PMC2826839

[180] Smith C, Carney JM, Starke-Reed P, Oliver C, Stadtman E, Floyd R, et al (1991). Excess brain protein oxidation and enzyme dysfunction in normal aging and in Alzheimer disease. Proceedings of the National Academy of Sciences, 88:10540-10543. 10.1073/pnas.88.23.10540 PMC52964 1683703

[181] Lyras L, Cairns NJ, Jenner A, Jenner P, Halliwell B (1997). An assessment of oxidative damage to proteins, lipids, and DNA in brain from patients with Alzheimer's disease. Journal of neurochemistry, 68:2061-2069. 9109533 10.1046/j.1471-4159.1997.68052061.x

[182] Boyd-Kimball D, Sultana R, Poon HF, Lynn BC, Casamenti F, Pepeu G, et al (2005). Proteomic identification of proteins specifically oxidized by intracerebral injection of amyloid beta-peptide (1-42) into rat brain: implications for Alzheimer's disease. Neuroscience, 132:313-324. 15802185 10.1016/j.neuroscience.2004.12.022

[183] Monnier VM, Mustata GT, Biemel KL, Reihl O, Lederer MO, Zhenyu D, et al (2005). Cross-linking of the extracellular matrix by the maillard reaction in aging and diabetes: an update on "a puzzle nearing resolution". Ann N Y Acad Sci, 1043:533-544. 16037276 10.1196/annals.1333.061

[184] Glenn JV, Mahaffy H, Wu K, Smith G, Nagai R, Simpson DA, et al (2009). Advanced glycation end product (AGE) accumulation on Bruch's membrane: links to age-related RPE dysfunction. Invest Ophthalmol Vis Sci, 50:441-451. 18676633 10.1167/iovs.08-1724

[185] Greenwald SE (2007). Ageing of the conduit arteries. J Pathol, 211:157-172.17200940

[186] Bartling B, Desole M, Rohrbach S, Silber RE, Simm A (2009). Age-associated changes of extracellular matrix collagen impair lung cancer cell migration. Faseb j, 23:1510-1520. 19109409 10.1096/fj.08-122648

[187] DeGroot J, Verzijl N, Budde M, Bijlsma JW, Lafeber FP, TeKoppele JM (2001). Accumulation of advanced glycation end products decreases collagen turnover by bovine chondrocytes. Exp Cell Res, 266:303-310. 11399058 10.1006/excr.2001.5224

[188] Smith MA, Taneda S, Richey PL, Miyata S, Yan S-D, Stern D, et al (1994). Advanced Maillard reaction end products are associated with Alzheimer disease pathology. Proceedings of the National Academy of Sciences, 91:5710-5714. 10.1073/pnas.91.12.5710 PMC44066 8202552

[189] Vicente Miranda H, Gomes MA, Branco-Santos J, Breda C, Lázaro DF, Lopes LV, et al (2016). Glycation potentiates neurodegeneration in models of Huntington’s disease. Scientific Reports, 6:36798. 27857176 10.1038/srep36798 PMC5114697

[190] Vicente Miranda H, El-Agnaf OM, Outeiro TF (2016). G lycation in P arkinson's disease and Alzheimer's disease. Movement Disorders, 31:782-790. 26946341 10.1002/mds.26566

[191] Li J, Liu D, Sun L, Lu Y, Zhang Z (2012). Advanced glycation end products and neurodegenerative diseases: mechanisms and perspective. J Neurol Sci, 317:1-5. 22410257 10.1016/j.jns.2012.02.018

[192] Ahmad S, Moinuddin, Dixit K, Shahab U, Alam K, Ali A (2011). Genotoxicity and immunogenicity of DNA-advanced glycation end products formed by methylglyoxal and lysine in presence of Cu2+. Biochem Biophys Res Commun, 407:568-574. 21420380 10.1016/j.bbrc.2011.03.064

[193] Vaca CE, Fang J-L, Conradi M, Hou S-M (1994). Development of a 32P-postlabelling method for the analysis of 2'-deoxyguanosine-3'-monophosphate and DNA adducts of methylglyoxal. Carcinogenesis, 15:1887-1894. 7923582 10.1093/carcin/15.9.1887

[194] Murata-Kamiya N, Kaji H, Kasai H (1999). Deficient nucleotide excision repair increases base-pair substitutions but decreases TGGC frameshifts induced by methylglyoxal in Escherichia coli. Mutation Research/Genetic Toxicology and Environmental Mutagenesis, 442:19-28. 10.1016/s1383-5718(99)00054-6 10366769

[195] Richarme G, Liu C, Mihoub M, Abdallah J, Leger T, Joly N, et al (2017). Guanine glycation repair by DJ-1/Park7 and its bacterial homologs. Science, 357:208. 28596309 10.1126/science.aag1095

[196] Koch M, Chitayat S, Dattilo BM, Schiefner A, Diez J, Chazin WJ, et al (2010). Structural basis for ligand recognition and activation of RAGE. Structure, 18:1342-1352. 20947022 10.1016/j.str.2010.05.017 PMC4250094

[197] Sessa L, Gatti E, Zeni F, Antonelli A, Catucci A, Koch M, et al (2014). The receptor for advanced glycation end-products (RAGE) is only present in mammals, and belongs to a family of cell adhesion molecules (CAMs). PloS one, 9:e86903. 24475194 10.1371/journal.pone.0086903 PMC3903589

[198] Khan MI, Rath S, Adhami VM, Mukhtar H (2017). Hypoxia driven glycation: Mechanisms and therapeutic opportunities. Semin Cancer Biol. 10.1016/j.semcancer.2017.05.008 PMC5699980 28546110

[199] Yamagishi S-i, Nakamura N, Suematsu M, Kaseda K, Matsui T (2015). Advanced Glycation End Products: A Molecular Target for Vascular Complications in Diabetes. Molecular Medicine, 21:S32-S40. 26605646 10.2119/molmed.2015.00067 PMC4661053

[200] Baynes JW, Watkins NG, Fisher CI, Hull CJ, Patrick JS, Ahmed MU, et al (1989). The Amadori product on protein: structure and reactions. Prog Clin Biol Res, 304:43-67. 2675036

[201] Li JJ, Voisin D, Quiquerez AL, Bouras C (1994). Differential expression of advanced glycosylation end-products in neurons of different species. Brain Res, 641:285-288. 8012830 10.1016/0006-8993(94)90156-2

[202] Ko SY, Lin YP, Lin YS, Chang SS (2010). Advanced glycation end products enhance amyloid precursor protein expression by inducing reactive oxygen species. Free Radic Biol Med, 49:474-480. 20471471 10.1016/j.freeradbiomed.2010.05.005

[203] Li YM, Dickson DW (1997). Enhanced binding of advanced glycation endproducts (AGE) by the ApoE4 isoform links the mechanism of plaque deposition in Alzheimer's disease. Neurosci Lett, 226:155-158. 9175590 10.1016/s0304-3940(97)00266-8

[204] Li XH, Lv BL, Xie JZ, Liu J, Zhou XW, Wang JZ (2012). AGEs induce Alzheimer-like tau pathology and memory deficit via RAGE-mediated GSK-3 activation. Neurobiol Aging, 33:1400-1410. 21450369 10.1016/j.neurobiolaging.2011.02.003

[205] Thornalley PJ, Langborg A, Minhas HS (1999). Formation of glyoxal, methylglyoxal and 3-deoxyglucosone in the glycation of proteins by glucose. Biochemical Journal, 344:109-116. 10548540 PMC1220620

[206] Turk Z (2010). Glycotoxines, carbonyl stress and relevance to diabetes and its complications. Physiol Res, 59:147-156. 19537931 10.33549/physiolres.931585

[207] Wautier JL, Guillausseau PJ (1998). Diabetes, advanced glycation endproducts and vascular disease. Vasc Med, 3:131-137. 9796076 10.1177/1358836X9800300207

[208] Kikuchi S, Shinpo K, Takeuchi M, Yamagishi S, Makita Z, Sasaki N, et al (2003). Glycation--a sweet tempter for neuronal death. Brain Res Brain Res Rev, 41:306-323. 12663085 10.1016/s0165-0173(02)00273-4

[209] Kraus LM, Kraus AP Jr, (2001). Carbamoylation of amino acids and proteins in uremia. Kidney Int Suppl, 78:S102-107. 11168993 10.1046/j.1523-1755.2001.59780102.x

[210] Jaisson S, Lorimier S, Ricard-Blum S, Sockalingum GD, Delevallee-Forte C, Kegelaer G, et al (2006). Impact of carbamylation on type I collagen conformational structure and its ability to activate human polymorphonuclear neutrophils. Chem Biol, 13:149-159. 16492563 10.1016/j.chembiol.2005.11.005

[211] Wang Z, Nicholls SJ, Rodriguez ER, Kummu O, Horkko S, Barnard J, et al (2007). Protein carbamylation links inflammation, smoking, uremia and atherogenesis. Nat Med, 13:1176-1184. 17828273 10.1038/nm1637

[212] Jaisson S, Larreta-Garde V, Bellon G, Hornebeck W, Garnotel R, Gillery P (2007). Carbamylation differentially alters type I collagen sensitivity to various collagenases. Matrix Biol, 26:190-196. 17156988 10.1016/j.matbio.2006.10.008

[213] Jaisson S, Kerkeni M, Santos-Weiss IC, Addad F, Hammami M, Gillery P (2015). Increased serum homocitrulline concentrations are associated with the severity of coronary artery disease. Clin Chem Lab Med, 53:103-110. 25153409 10.1515/cclm-2014-0642

[214] Apostolov EO, Basnakian AG, Ok E, Shah SV (2012). Carbamylated low-density lipoprotein: nontraditional risk factor for cardiovascular events in patients with chronic kidney disease. J Ren Nutr, 22:134-138. 22200430 10.1053/j.jrn.2011.10.023

[215] Gorisse L, Pietrement C, Vuiblet V, Schmelzer CEH, Köhler M, Duca L, et al (2016). Protein carbamylation is a hallmark of aging. Proceedings of the National Academy of Sciences of the United States of America, 113:1191-1196. 26712018 10.1073/pnas.1517096113 PMC4747743

[216] Fando J, Grisolia S (1974). Carbamylation of brain proteins with cyanate in vitro and in vivo. Eur J Biochem, 47:389-396. 4412303 10.1111/j.1432-1033.1974.tb03704.x

[217] Crist RD, Grisolia S, Bettis CJ, Grisolia J (1973). Carbamoylation of proteins following administration to rats of carbamoyl phosphate and cyanate and effects on memory. Eur J Biochem, 32:109-116. 4687386 10.1111/j.1432-1033.1973.tb02585.x

[218] Ok E, Basnakian AG, Apostolov EO, Barri YM, Shah SV (2005). Carbamylated low-density lipoprotein induces death of endothelial cells: a link to atherosclerosis in patients with kidney disease. Kidney Int, 68:173-178. 15954906 10.1111/j.1523-1755.2005.00391.x

[219] Apostolov EO, Basnakian AG, Yin X, Ok E, Shah SV (2007). Modified LDLs induce proliferation-mediated death of human vascular endothelial cells through MAPK pathway. American Journal of Physiology - Heart and Circulatory Physiology, 292:H1836-H1846. 10.1152/ajpheart.01079.2006 17158646

[220] Gajjala PR, Fliser D, Speer T, Jankowski V, Jankowski J (2015). Emerging role of post-translational modifications in chronic kidney disease and cardiovascular disease. Nephrology Dialysis Transplantation, 30:1814-1824. 10.1093/ndt/gfv048 25862763

[221] Wong CM, Marcocci L, Liu L, Suzuki YJ (2010). Cell signaling by protein carbonylation and decarbonylation. Antioxidants & redox signaling, 12:393-404. 19686045 10.1089/ars.2009.2805 PMC2823370

[222] Tyler-Cross R, Schirch V (1991). Effects of amino acid sequence, buffers, and ionic strength on the rate and mechanism of deamidation of asparagine residues in small peptides. Journal of Biological Chemistry, 266:22549-22556. 1939272

[223] Robinson AB (1974). Evolution and the Distribution of Glutaminyl and Asparaginyl Residues in Proteins. Proceedings of the National Academy of Sciences, 71:885-888. 10.1073/pnas.71.3.885 PMC388120 4522799

[224] McKerrow JH, Robinson AB (1974). Primary Sequence Dependence of the Deamidation of Rabbit Muscle Aldolase. Science, 183:85-85. 4808790 10.1126/science.183.4120.85

[225] Scotchler JW, Robinson AB (1974). Deamidation of glutaminyl residues: dependence on pH, temperature, and ionic strength. Anal Biochem, 59:319-322. 4407737 10.1016/0003-2697(74)90040-2

[226] Robinson AB, Robinson LR (1991). Distribution of glutamine and asparagine residues and their near neighbors in peptides and proteins. Proceedings of the National Academy of Sciences, 88:8880-8884. 10.1073/pnas.88.20.8880 PMC52614 1924347

[227] Robinson AB, McKerrow JH, Cary P (1970). Controlled Deamidation of Peptides and Proteins: An Experimental Hazard and a Possible Biological Timer. Proceedings of the National Academy of Sciences, 66:753-757. 10.1073/pnas.66.3.753 PMC283114 5269237

[228] Robinson N, Robinson A, Merrifield R (2001). Mass spectrometric evaluation of synthetic peptides as primary structure models for peptide and protein deamidation. Chemical Biology & Drug Design, 57:483-493. 10.1034/j.1399-3011.2001.00863.x 11437952

[229] Hao P, Ren Y, Alpert AJ, Sze SK (2011). Detection, evaluation and minimization of nonenzymatic deamidation in proteomic sample preparation. Mol Cell Proteomics, 10:O111.009381. 10.1074/mcp.O111.009381 PMC3205879 21784994

[230] Hao P, Ren Y, Datta A, Tam JP, Sze SK (2015). Evaluation of the effect of trypsin digestion buffers on artificial deamidation. J Proteome Res, 14:1308-1314. 25495137 10.1021/pr500903b

[231] Hao P, Sze SK (2014). Proteomic analysis of protein deamidation. Curr Protoc Protein Sci, 78:24. 25 21-14. 10.1002/0471140864.ps2405s78 25367007

[232] Hao P, Qian J, Dutta B, Cheow ESH, Sim KH, Meng W, et al (2012). Enhanced Separation and Characterization of Deamidated Peptides with RP-ERLIC-Based Multidimensional Chromatography Coupled with Tandem Mass Spectrometry. Journal of Proteome Research, 11:1804-1811. 22239700 10.1021/pr201048c

[233] Serra A, Gallart-Palau X, Wei J, Sze SK (2016). Characterization of Glutamine Deamidation by Long-Length Electrostatic Repulsion-Hydrophilic Interaction Chromatography-Tandem Mass Spectrometry (LERLIC-MS/MS) in Shotgun Proteomics. Analytical Chemistry, 88:10573-10582. 27689507 10.1021/acs.analchem.6b02688

[234] Dutta B, Park JE, Kumar S, Hao P, Gallart-Palau X, Serra A, et al (2017). Monocyte adhesion to atherosclerotic matrix proteins is enhanced by Asn-Gly-Arg deamidation. Sci Rep, 7:5765. 28720870 10.1038/s41598-017-06202-2 PMC5515959

[235] Groenen PJ, Merck KB, de Jong WW, Bloemendal H (1994). Structure and modifications of the junior chaperone alpha-crystallin. From lens transparency to molecular pathology. Eur J Biochem, 225:1-19. 7925426 10.1111/j.1432-1033.1994.00001.x

[236] Harrington V, McCall S, Huynh S, Srivastava K, Srivastava OP (2004). Crystallins in water soluble-high molecular weight protein fractions and water insoluble protein fractions in aging and cataractous human lenses. Mol Vis, 10:476-489. 15303090

[237] Takata T, Lampi KJ, Oxford JT, Demeler B (2008). Deamidation destabilizes and triggers aggregation of a lens protein, beta A3-crystallin. Protein science, 17:1565-1575. 18567786 10.1110/ps.035410.108 PMC2525517

[238] Van Kleef FS, De Jong WW, Hoenders HJ (1975). Stepwise degradations and deamidation of the eye lens protein alpha-crystallin in ageing. Nature, 258:264-266. 1202360 10.1038/258264a0

[239] Sharma KK, Santhoshkumar P (2009). Lens Aging: Effects of Crystallins. Biochimica et biophysica acta, 1790:1095-1108. 19463898 10.1016/j.bbagen.2009.05.008 PMC2743770

[240] Lindner H, Sarg B, Grunicke H, Helliger W (1999). Age-dependent deamidation of H1(0) histones in chromatin of mammalian tissues. J Cancer Res Clin Oncol, 125:182-186. 10235472 10.1007/s004320050261 PMC12199871

[241] Ghosh AK, Osswald HL (2014). BACE1 (beta-secretase) inhibitors for the treatment of Alzheimer's disease. Chem Soc Rev, 43:6765-6813. 24691405 10.1039/c3cs60460h PMC4159447

[242] De Strooper B, Chavez Gutierrez L (2015). Learning by failing: ideas and concepts to tackle gamma-secretases in Alzheimer's disease and beyond. Annu Rev Pharmacol Toxicol, 55:419-437. 25292430 10.1146/annurev-pharmtox-010814-124309

[243] Sitia R, Palladini G, Merlini G (2007). Bortezomib in the treatment of AL amyloidosis: targeted therapy? Haematologica, 92:1302-1307. 18024367 10.3324/haematol.12136

[244] Stangou AJ, Banner NR, Hendry BM, Rela M, Portmann B, Wendon J, et al (2010). Hereditary fibrinogen A alpha-chain amyloidosis: phenotypic characterization of a systemic disease and the role of liver transplantation. Blood, 115:2998-3007. 19633201 10.1182/blood-2009-06-223792

[245] Yiannopoulou KG, Papageorgiou SG (2013). Current and future treatments for Alzheimer's disease. Therapeutic advances in neurological disorders, 6:19-33. 23277790 10.1177/1756285612461679 PMC3526946

[246] Baazaoui N, Iqbal K (2018). A Novel Therapeutic Approach to Treat Alzheimer's Disease by Neurotrophic Support During the Period of Synaptic Compensation. Journal of Alzheimer's disease : JAD, 62:1211-1218. 29562539 10.3233/JAD-170839 PMC5870029

[247] Farlow M (2002). A clinical overview of cholinesterase inhibitors in Alzheimer's disease. Int Psychogeriatr, 14 Suppl 1:93-126. 12636182 10.1017/s1041610203008688

[248] Jayalakshmi K, Sairam M, Singh SB, Sharma SK, Ilavazhagan G, Banerjee PK (2005). Neuroprotective effect of N-acetyl cysteine on hypoxia-induced oxidative stress in primary hippocampal culture. Brain Res, 1046:97-104. 15919066 10.1016/j.brainres.2005.03.054

[249] Reddy VP, Mehta J, Aliev G 2015 Chapter 88 - Role of α-Lipoid Acid and Acetyl-L-Carnitine in Dementia A2 - Martin Colin R In Diet and Nutrition in Dementia and Cognitive Decline. Preedy VR, editor. San Diego: Academic Press 955-962.

